# Occupational burnout in women schoolteachers in Kazakhstan: psychometric evaluation and psychosocial correlates within the job demands-resources framework

**DOI:** 10.3389/fpsyg.2026.1945234

**Published:** 2026-09-29

**Authors:** Nurlan Smagulov, Olzhas Zhamantayev, Ayaulym Arystanbay, Tatiana Lysakova, Dmitry Ageev, Gulzhazira Turlybekova

**Affiliations:** 1 Faculty of Biology and Geography, Buketov Karaganda National Research University, Karaganda, Kazakhstan; 2 School of Public Health, Karaganda Medical University, Karaganda, Kazakhstan; 3 Faculty of Mathematics and Natural Sciences, Kozybayev University, Petropavlovsk, Kazakhstan

**Keywords:** emotional exhaustion, gender, job demands-resources model, Kazakhstan, occupational burnout, subjective wellbeing, women teachers

## Abstract

**Background:**

Teaching combines intense interpersonal demands with administrative and digital workload, and in Kazakhstan more than four fifths of schoolteachers are women, which makes burnout in this group a gender-relevant occupational health and wellbeing concern. Evidence from Central Asia remains scarce, and the performance of the Russian-language Maslach Burnout Inventory-Educators Survey (MBI-ES) in this population had not been formally examined.

**Methods:**

In a cross-sectional study conducted between October and December 2025, 323 women teachers recruited by convenience in general education schools in three Kazakhstani cities completed the MBI-ES, the state scale of the Spielberger State–Trait Anxiety Inventory, and a structured questionnaire covering employment, workload, social-psychological, lifestyle, and digital factors. We split the sample at random, examined the structure in one half and confirmed it in the other, compared the three-factor model against one-factor, two-factor, and bifactor alternatives, tested measurement invariance, and fitted hierarchical regression models built on the job demands-resources framework.

**Results:**

The correlated three-factor model outperformed one-factor and two-factor alternatives and fitted the validation subsample well (*χ*^2^/df = 1.69; CFI = 0.955; RMSEA = 0.065; SRMR = 0.080). A bifactor model fitted better on global indices but yielded a degenerate exhaustion-specific factor and negative general loadings for accomplishment items, so we retained three factors. Loadings and intercepts were invariant across the random halves; across cities loadings held but intercepts did not. Subscale reliability was high (α 0.918 to 0.963). Job demands added 35.1% of explained variance in emotional exhaustion beyond background characteristics, resources a further 6.6%; perceived work stress, state anxiety, and low physical activity remained independent correlates in the full model (adjusted *R*^2^ = 0.630). Depersonalization was associated with conflict, work stress, isolation, and an unfavorable collegial climate (adjusted *R*^2^ = 0.443), and personal accomplishment gave the weakest model (adjusted *R*^2^ = 0.170). Demographic and formal workload characteristics contributed little.

**Conclusion:**

The Russian-language MBI-ES performs acceptably in women schoolteachers in Kazakhstan. In this convenience sample, burnout was associated more closely with appraised psychosocial working conditions than with formal job parameters. The cross-sectional design supports no causal inference, and the findings apply to women teachers in three urban settings.

## 1 Introduction

The teaching profession occupies a structurally strained position within occupational health. It combines the relational intensity typical of human services with administrative accountability pressures and the cognitive demands generated by digital educational systems. When such combined load persists without adequate recovery, it produces occupational burnout, a syndrome of emotional exhaustion, cynical detachment from professional relationships, and a diminished sense of personal effectiveness (Maslach and Jackson, 1981; Maslach et al., 2001). The World Health Organization classifies burnout as an occupational phenomenon arising from chronically unmanaged workplace stress (WHO, 2019). Burnout is not a transient stress reaction but a chronic adaptive failure with measurable consequences for teachers’ physical and mental health, instructional quality, and workforce stability (Salvagioni et al., 2017; Maslach and Leiter, 2016; Madigan and Kim, 2021).

The scale of the problem has been documented across national contexts. A scoping review of the international literature documented wide variation in the prevalence of teacher stress, burnout, anxiety, and depression across countries and instruments (Agyapong et al., 2022). Elevated emotional exhaustion appears in most European teacher studies (Iriarte Redín and Erro-Garcés, 2020), reviews indicate that burnout rose during the pandemic period (Westphal et al., 2022), and a meta-analysis of nine pandemic-period studies estimated a pooled burnout prevalence of 52% (95% CI 33–71%), higher than the rates reported for health professionals and higher among women than men (Ozamiz-Etxebarria et al., 2023). Burnout is among the leading correlates of turnover intention and attrition in the education sector (Madigan and Kim, 2021), a pattern consistent with TALIS data, in which workload and administrative tasks rank among the leading reported sources of teacher stress and these demands are linked to wellbeing and intentions to leave the profession (OECD, 2020, 2025).

The job demands-resources (JD-R) model supplies the theoretical frame for this study. First proposed by Demerouti et al. (2001) and elaborated by Bakker and Demerouti (2007) and Bakker et al. (2023), the model holds that burnout develops when job demands chronically exceed the personal and organizational resources available to meet them. Demands drive an energy-depletion process expressed primarily as emotional exhaustion, whereas resource deficits drive a motivational-withdrawal process expressed as depersonalization. This dual-pathway logic has received broad empirical support in teacher samples across European, North American, and East Asian settings (Hakanen et al., 2006; Alarcon, 2011; Skaalvik and Skaalvik, 2017, 2018), including a full longitudinal test of the model’s assumptions in 1,700 beginning teachers (Dicke et al., 2018) and a multilevel study of more than 10,000 preschool teachers in China in which job resources related to lower and job demands to higher burnout (Zhao et al., 2023).

The three-dimensional construct assessed here follows Maslach and Jackson (1981) as operationalized in the MBI-ES. Emotional exhaustion (EE) reflects the depletion of emotional and energetic resources under prolonged interpersonal and cognitive load and represents the most direct response to chronic occupational strain. Depersonalization (DP) reflects the development of a detached, cynical stance toward the recipients of one’s work, understood as a secondary defensive adaptation to exhaustion (Lee and Ashforth, 1996). Reduced personal accomplishment (PA) reflects a progressive decline in perceived professional competence, a dimension governed partly by appraisal and attribution processes that operate somewhat independently of the strain pathway (Schaufeli et al., 2009). Among the most consistently replicated correlates of EE are perceived occupational stress and anxiety: emotional demands and overload carry the largest pooled effects on EE (Alarcon, 2011), subjective stress is the most consistent EE correlate across countries (Agyapong et al., 2022), anxiety amplifies the impact of job demands while impairing recovery (Kidger et al., 2016; Koutsimani et al., 2019; Hooker et al., 2025). A systematic review of K-12 teachers indicates that they may carry higher levels of depression and anxiety than the general population, with perceived stress and poor school climate among the associated factors (Hooker et al., 2025). Interpersonal conflict is among the more specific antecedents of DP, activating defensive distancing in response to relational threat (Leiter and Maslach, 2004), while perceived social isolation compounds this risk through a resource-conservation mechanism (Halbesleben, 2006). A positive collegial climate, by contrast, buffers DP as an organizational resource (Collie et al., 2012; Nwoko et al., 2023; Gao and Wang, 2026). The accelerated integration of digital tools into schooling has produced technostress, the strain arising when workers cannot adapt to technology at the pace institutions require, a condition linked to exhaustion and lowered professional self-efficacy (Tarafdar et al., 2015; La Torre et al., 2019; Califf and Brooks, 2020; Fernández-Batanero et al., 2021); the pressure for constant digital availability has emerged as an added burden of the same kind, predicting burnout among schoolteachers (Qin et al., 2025). Physical activity, in turn, functions as a recovery-supporting resource that promotes detachment from work demands and relates to lower burnout within the JD-R framework itself (Sonnentag and Fritz, 2015; Fodor et al., 2020; Xu et al., 2025).

Between 2012 and 2025, the Kazakhstani school system underwent structural transformations that substantially altered teachers’ working conditions: an updated national curriculum expanded formative-assessment and documentation duties, the trilingual education policy created new language-training demands, and the mass adoption of digital platforms during the pandemic required rapid competence development in a workforce whose digital pedagogical preparation was limited and uneven (Karabassova, 2020; OECD, 2023). Reform-related stress has been identified as a key determinant of turnover intentions among Kazakhstani teachers in TALIS data (Nurmukhamed et al., 2025).

The present study examines women teachers specifically, and this choice carries substantive rather than merely methodological weight. Women account for 82.1% of the pedagogical workforce in Kazakhstan (Ministry of Education of the Republic of Kazakhstan, 2025), a degree of feminization typical of the profession internationally. Meta-analytic evidence indicates that women report higher emotional exhaustion than men (Purvanova and Muros, 2010), and longitudinal Swedish data show that women teachers carry heavier emotional and quantitative demands at work together with a larger share of unpaid domestic work (Stengård et al., 2022). Most teacher burnout research is conducted, de facto, on predominantly female samples (Agyapong et al., 2022), and studies restricted to women teachers are an established design in the field (Tokarskaya et al., 2025). Within an equity-oriented wellbeing perspective, burnout in a feminized, emotionally laborious, and reform-burdened occupation is a marker of structural vulnerability, not only an individual outcome. Restricting the sample to women also avoided confounding gender with subject specialization, since male teachers in the surveyed schools were concentrated in physical education, technology, and pre-conscription training.

Cross-cultural transferability of the MBI is an empirical question rather than an assumption. Validation studies have supported the three-factor structure in European, African, East Asian, and Latin American occupational groups (Worley et al., 2008; Aguayo et al., 2011; Szigeti et al., 2017; Aboagye et al., 2018; Ntouka et al., 2026). Alternative structures nonetheless keep appearing: a bifactor solution fitted best in a random sample of 1,206 Polish primary school teachers, where the oblique three-factor model was not supported (Hawrot and Koniewski, 2018); a two-factor model combining exhaustion and depersonalization was favored in a Bayesian reanalysis of 5,575 French teachers (De Beer and Bianchi, 2019); and a recent four-country study of the MBI-ES among early childhood professionals achieved partial scalar invariance only for a single exhaustion factor (Lemos et al., 2025). The PA subscale has shown the lowest and most variable performance in cross-cultural applications, with item-total correlations sometimes approaching zero in non-Western samples (Poghosyan et al., 2009). Systematic psychometric evaluation is thus a precondition for meaningful interpretation in any new linguistic and cultural context, and the instrument’s performance in post-Soviet Central Asian populations remains almost undocumented in indexed literature. To our knowledge, this is among the first studies in Kazakhstan to combine exploratory and confirmatory factor analysis of the Russian-language MBI-ES with multivariable modeling of organizational and psychosocial correlates in a homogeneous sample of women teachers.

The study pursued three aims: to test the three-factor structure of the Russian-language MBI-ES in women secondary school teachers in Kazakhstan; to characterize bivariate associations between occupational, psychoemotional, organizational, lifestyle, and digital-informational variables and the three burnout dimensions; and to estimate the independent contribution of these variables to EE, DP, and PA. Drawing on the JD-R model and prior evidence on the differential stability of the MBI components, we tested four hypotheses. H1: higher perceived job demands, indexed by perceived work stress, workload, and information overload, are associated with more pronounced emotional exhaustion. H2: relational stressors, specifically the frequency of interpersonal conflict and perceived social isolation, are associated with more pronounced depersonalization. H3: available resources, namely regular physical activity and a favorable interpersonal climate, are associated with lower burnout on the affected dimensions. H4: the personal accomplishment subscale shows lower and less stable psychometric performance than the emotional exhaustion and depersonalization subscales.

## 2 Materials and methods

### 2.1 Study design and participant selection

We conducted a cross-sectional observational study framed by the JD-R model (Bakker and Demerouti, 2007). Data were collected between October and December 2025, an inter-examination period of the school calendar with elevated but relatively stable professional demands. This window was chosen to reduce the risk of inflating burnout estimates through situational reactivity, since exhaustion and depersonalization scores tend to rise toward the end of the school year (Marić et al., 2022). Recruitment proceeded by convenience through school administrations in the three cities. Within each participating school, all teachers present on the survey day who met the inclusion criteria were invited, so sampling within schools was exhaustive rather than selective. Questionnaires were distributed in person, completed on site, and returned independently of school management. The analytic sample was distributed as 132 participants in Astana (40.9%), 101 in Petropavlovsk (31.3%), and 90 in Karaganda (27.9%).

To preserve anonymity, the questionnaire carried no school identifier, and responses cannot be linked to the school in which they were collected. We are unable to quantify clustering within schools or to fit models with a school-level random effect, and all analyses treat observations as independent. Section 4.4 returns to this constraint.

### 2.2 Participants

In total, 359 teachers consented and completed the questionnaire. To keep the analytic sample methodologically homogeneous and to avoid confounding gender with subject specialization, the 36 male respondents (10.0%) were excluded, leaving 323 women teachers. This decision reflects the pronounced feminization of the profession in Kazakhstan, where women make up 82.1% of the pedagogical workforce (484,788 persons), including 334,641 employed in general secondary education (Ministry of Education of the Republic of Kazakhstan, 2025). Male respondents were concentrated in physical education, technology, and pre-conscription training, disciplines that together accounted for only 7.7% of the sample, so their inclusion would have created small, professionally heterogeneous subgroups incomparable in specialization, size, and age structure. Inclusion criteria were current employment as a teacher in a state general education school, voluntary informed participation, and absence of acute illness at the time of the survey. Holding a primarily non-teaching position, acute illness, or refusal constituted exclusion criteria.

### 2.3 Ethical considerations

The study was approved by the local bioethics committee of Karaganda Medical University, Karaganda, Republic of Kazakhstan (Protocol No. 17, 22 October 2024). All procedures followed the Declaration of Helsinki and national legislation on research involving human participants. Participants received a written information sheet describing the aim of the study, the voluntary nature of participation, and the right to withdraw at any stage without consequences. Because the survey was fully anonymous, the committee approved implied informed consent, whereby completion and return of the questionnaire confirmed willingness to participate.

### 2.4 Measures

Occupational burnout was assessed with the Russian-language Maslach Burnout Inventory-Educators Survey (MBI-ES; Maslach and Jackson, 1981; Maslach et al., 1996), the most widely used burnout measure in teacher research, with cross-cultural validation in numerous national contexts (Aguayo et al., 2011; Aboagye et al., 2018; Ntouka et al., 2026). Although newer instruments exist, the MBI remains the reference measure in international teacher studies, systematic reviews, and meta-analyses, and its use preserves comparability with the accumulated evidence base, which was central to the psychometric aim of this study. The instrument was administered under license from Mind Garden, Inc. (Menlo Park, CA, United States), which holds the copyright and publishes the MBI-ES. No item of the instrument is reproduced in this article or in its Supplementary material. We administered the Russian-language adaptation of the MBI standardized by Vodopyanova et al. (2013), with the educator wording of the item stems. This version follows the item content, subscale composition, and response format of the MBI-ES, and the psychometric results reported below apply to it rather than to any other Russian rendering of the instrument. The MBI-ES contains 22 items in three subscales: emotional exhaustion (EE, nine items), depersonalization (DP, five items), and personal accomplishment (PA, eight items, interpreted inversely), each rated on a seven-point frequency scale from 0 (never) to 6 (every day). Subscale scores were computed as item sums (theoretical ranges: EE 0–54; DP 0–30; PA 0–48).

State (situational) anxiety was measured with the state scale of the Spielberger State–Trait Anxiety Inventory (STAI-S; Spielberger, 1983) in the Russian adaptation by Khanin (1976), which is standard in Russian-language educational research. Raw STAI-S scores (theoretical range 20–80; observed 20–74) were retained for all analyses.

A structured questionnaire collected sociodemographic characteristics (age, length of service, marital status, number of children) and variables spanning occupational status, teaching and total workload, administrative and documentation load, social-psychological conditions, lifestyle, and digital-informational aspects of teachers’ work, constructed on the basis of the JD-R model and the technostress literature (Tarafdar et al., 2015; La Torre et al., 2019). Supplementary Table S1 presents the full codebook of the study variables (X1-X22) with response categories and theoretical domain.

### 2.5 Statistical analysis

Distributional properties were screened with skewness and kurtosis coefficients and with Kolmogorov–Smirnov and Shapiro–Wilk tests. Formal tests indicated significant departures from normality for most variables (*p* < 0.05), an expected outcome at n > 300 given their sensitivity to trivial deviations; following Kim (2013), absolute skewness below 2 and kurtosis below 7, which held for all variables (maximum skewness 1.58, maximum kurtosis 2.14), was taken to permit parametric factor-analytic and regression procedures, while rank-based statistics were used for bivariate analysis.

Internal consistency was evaluated separately for each MBI subscale with Cronbach’s α, McDonald’s ω, corrected item-total correlations (criterion ≥ 0.30), and α-if-item-deleted values (Tavakol and Dennick, 2011).

To separate exploration from confirmation, we split the sample at random into two halves stratified by study site, using a fixed seed for reproducibility. The calibration half (*n* = 161) served for exploratory factor analysis, and the validation half (*n* = 162) for confirmatory analysis. All exploratory procedures were repeated in the full sample for comparability with earlier MBI validation reports.

The latent structure was examined with principal axis factoring and oblique Promax rotation with Kaiser normalization, since the burnout components are theoretically and empirically correlated. Sampling adequacy was checked with the Kaiser-Meyer-Olkin coefficient and Bartlett’s test of sphericity, the number of factors was set by the Kaiser criterion (eigenvalue ≥ 1.0) with scree-plot inspection (Kaiser, 1974), and rotated loadings ≥ 0.70 were read as strong indicator-factor links (Hair et al., 2019). Composite reliability (CR) and average variance extracted (AVE) were computed from the pattern loadings, with CR ≥ 0.70 and AVE ≥ 0.50 as thresholds. Discriminant validity was assessed with the Fornell-Larcker criterion and with heterotrait-monotrait (HTMT) ratios, using 0.85 as the reference value.

Confirmatory factor analysis was estimated by maximum likelihood in IBM SPSS AMOS. Rather than testing the canonical structure in isolation, we compared four competing measurement models: a single-factor model, a two-factor model in which exhaustion and depersonalization items load on one factor and accomplishment items on a second, the correlated three-factor model, and a bifactor model with a general factor and three orthogonal specific factors. Model fit was judged on absolute, comparative, and approximation indices (*χ*^2^/df ≤ 3.0, GFI and AGFI ≥ 0.90, TLI, NFI, and CFI ≥ 0.90, RMSEA ≤ 0.08 with its 90% confidence interval, SRMR ≤ 0.08; Hu and Bentler, 1999; Kline, 2016) together with AIC and BIC, and nested models were compared by the likelihood ratio test. Because global fit indices favor more heavily parameterized solutions, the bifactor model was evaluated on the interpretability of its loadings and on hierarchical omega, subscale hierarchical omega, explained common variance, and the percentage of uncontaminated correlations, rather than on fit alone. Local fit was examined through the matrix of residual correlations. All confirmatory analyses were run in the validation half and repeated in the full sample.

Measurement invariance was tested by multi-group analysis across the two random halves and across the three study sites, in the sequence configural, metric, scalar. Judgments rested on the likelihood ratio test together with ΔCFI ≤ 0.010 and ΔRMSEA ≤ 0.015.

Bivariate associations between the study variables and the three burnout dimensions were estimated with Spearman’s rank correlation (ρ) and are reported for the full variable set.

Independent contributions were estimated with hierarchical multiple linear regression organized by the job demands-resources framework. Block 1 contained background and occupational characteristics (age, length of service, marital status, number of children, subject area, employment type). Block 2 contained job demands: quantitative workload (weekly teaching hours, total weekly working hours, administrative duties, documentation load), digital-informational demands (daily screen time, information overload), and relational demands (conflict frequency, perceived social isolation). Block 3 contained job and recovery resources (satisfaction with the interpersonal climate, appraisal of digitalization, sleep duration, physical activity, meal regularity). Block 4 contained appraised strain (perceived work stress, emotional tension, state anxiety), entered last because these constructs lie closest to the outcome and their placement allows the demand-related and resource-related variance to be read before they are absorbed. Nominal predictors (marital status, subject area) were dummy-coded. We report standardized coefficients (*β*) with 95% confidence intervals, *R*^2^, adjusted *R*^2^, Δ*R*^2^ with its F test at each step, and variance inflation factors.

Three sensitivity analyses accompany the hierarchical models: exclusion of length of service, which correlates with age at r = 0.90; adjustment for study site; and re-estimation of the exhaustion model without state anxiety, whose content overlaps with the outcome. For comparability with the wider burnout literature we also report exploratory stepwise models (probability of F to enter ≤ 0.05, to remove ≥ 0.10) over the pool of 20 variables (X3 to X22), and we quantify the stability of that procedure by repeating it in 500 bootstrap resamples and recording how often each predictor is retained. Model assumptions were checked with histograms, normal P–P plots, and residual-versus-predicted scatterplots of standardized residuals, and residual independence with the Durbin-Watson statistic (Field, 2018).

Common method variance was addressed with the Harman single-factor test computed across all 36 self-report variables, with the confirmatory single-factor model, and with a marker-variable adjustment using the smallest observed association between a questionnaire variable and a burnout score as the marker estimate (Lindell and Whitney, 2001). Data preparation was performed in Microsoft Excel 2019, and all analyses were run in IBM SPSS Statistics 26 with confirmatory models in AMOS.

## 3 Results

### 3.1 Sample characteristics

The analytic sample comprised 323 women teachers (Table 1). Mean age was 42.10 ± 11.98 years and mean length of service 18.36 ± 12.28 years. The largest age group was 50–59 years (*n* = 86; 26.6%), and 40.2% of participants (*n* = 130) had more than 20 years of service, indicating a workforce weighted toward the late career stage. A quarter of the sample (*n* = 83; 25.7%) reported teaching above the full contractual load.

**Table 1:** Sociodemographic and occupational characteristics of the sample (*n* = 323).

Characteristic	Category	*n*	%
City	Astana	132	40.9
	Petropavlovsk	101	31.3
	Karaganda	90	27.9
Age group	< 30 years	56	17.3
	30–39 years	81	25.1
	40–49 years	80	24.8
	50–59 years	86	26.6
	≥ 60 years	20	6.2
Length of service	< 5 years	50	15.5
	5–10 years	67	20.7
	11–20 years	76	23.5
	> 20 years	130	40.2
Employment typeᵃ	Part-time	32	9.9
	Full-time	207	64.1
	Above full contract	83	25.7
Subject area	Primary grades	108	33.4
	STEM subjects	71	22.0
	Languages / humanities	119	36.8
	Arts / physical education / technology	25	7.7
Marital status	Single	62	19.2
	Married	206	63.8
	Divorced	36	11.1
	Widowed	19	5.9
Number of children	None	70	21.7
	One	73	22.6
	Two	103	31.9
	Three or more	77	23.8

ᵃOne record contained an out-of-range employment code and was treated as missing (valid *n* = 322).

Supplementary Table S1 gives the coding of all study variables. In absolute terms the reported workload was moderate, since only 8.7% (*n* = 28) placed their total weekly working time above 40 h and 19.2% (*n* = 62) taught more than 24 h a week. Duties beyond the classroom were far more widespread: 65.6% (*n* = 212) reported moderate or substantial administrative duties and 31.6% (*n* = 102) rated documentation and reporting load as high or excessive. Subjective strain followed a similar pattern. Work stress arose often or always for 31.0% (*n* = 100), and emotional tension was high or very high for 22.3% (*n* = 72). State anxiety averaged 36.22 ± 11.38 points (Md = 34; range 20–74), reaching the moderate band in 40.9% (*n* = 132) and the high band in 20.4% (*n* = 66) of participants.

The social environment of the schools appeared largely free of open conflict, since 54.2% (*n* = 175) reported no interpersonal conflicts over the year, yet the quality of collegial relations was mixed: only 31.3% (*n* = 101) rated the interpersonal climate as high or very high, and 29.1% (*n* = 94) felt socially isolated at least sometimes. Recovery resources were limited. More than half slept less than 7 h (54.8%; *n* = 177), 35.6% (*n* = 115) reported no regular physical activity, and 44.3% (*n* = 143) ate irregularly or skipped meals. Digital exposure was heavy, with 67.8% (*n* = 219) spending more than 4 h a day on screens and 46.7% (*n* = 151) experiencing information overload often or always, although 57.3% (*n* = 185) judged digitalization to simplify their work.

Observed burnout scores differed across sites. Emotional exhaustion was highest in Astana and lowest in Karaganda (means 26.6, 23.5, and 20.1 for Astana, Petropavlovsk, and Karaganda; Kruskal-Wallis *p* < 0.001), personal accomplishment ran in the opposite direction (lowest in Astana at 29.0, highest in Petropavlovsk at 34.0; *p* < 0.001), and depersonalization did not differ (*p* = 0.24). Study site was carried into the regression models as a sensitivity check (Section 3.6). Item intercepts were not invariant across sites (Section 3.4.1), so these are differences in observed scores alone. They do not establish that latent burnout differs between the three cities, and we do not interpret them as such at any point below.

### 3.2 Descriptive statistics of the MBI-ES and internal consistency

Mean subscale scores were 23.80 ± 11.34 for EE (observed range 0–52), 8.45 ± 5.95 for DP (0–27), and 31.51 ± 7.84 for PA (5–48). Emotional exhaustion was thus the most pronounced burnout component in this sample. Item means ranged 2.27–2.97 for EE, 1.59–1.85 for DP, and 3.74–4.24 for PA. All skewness (−0.51 to 0.61) and kurtosis (−0.72 to 0.18) values lay within accepted bounds (Table 2).

**Table 2:** MBI-ES item statistics and subscale reliability.

Item	M ± SD	Skewness	Kurtosis	Corrected item-total rᵃ	α if item deletedᵃ
EE subscale: α = 0.963					
EE1	2.72 ± 1.43	0.12	−0.17	0.821	0.959
EE2	2.97 ± 1.45	0.04	−0.52	0.860	0.958
EE3	2.46 ± 1.41	0.30	−0.26	0.838	0.959
EE4	2.95 ± 1.53	0.05	−0.61	0.855	0.958
EE5	2.27 ± 1.40	0.38	−0.44	0.872	0.957
EE6	2.43 ± 1.46	0.22	−0.53	0.868	0.957
EE7	2.77 ± 1.37	−0.05	−0.58	0.816	0.960
EE8	2.85 ± 1.49	0.14	−0.63	0.868	0.957
EE9	2.38 ± 1.36	0.10	−0.58	0.796	0.961
DP subscale: α = 0.956					
DP1	1.59 ± 1.26	0.61	−0.12	0.861	0.948
DP2	1.60 ± 1.29	0.57	−0.39	0.888	0.943
DP3	1.85 ± 1.34	0.41	−0.54	0.883	0.944
DP4	1.73 ± 1.20	0.34	−0.40	0.881	0.945
DP5	1.68 ± 1.36	0.55	−0.46	0.872	0.946
PA subscale: α = 0.918					
PA1	3.94 ± 1.17	−0.13	0.18	0.629	0.915
PA2	3.80 ± 1.14	−0.21	0.00	0.695	0.909
PA3	4.24 ± 1.31	−0.31	−0.67	0.749	0.905
PA4	4.14 ± 1.29	−0.51	−0.09	0.733	0.906
PA5	3.90 ± 1.21	−0.14	−0.49	0.748	0.905
PA6	3.74 ± 1.26	−0.05	−0.58	0.759	0.904
PA7	3.94 ± 1.24	−0.16	−0.44	0.759	0.904
PA8	3.81 ± 1.23	0.01	−0.47	0.742	0.906

𝐼omputed within each subscale. All items are keyed 0–6.

All three subscales showed high internal consistency: Cronbach’s α was 0.963 for EE, 0.956 for DP, and 0.918 for PA. McDonald’s ω took the same values to three decimals, which is expected when loadings within a subscale are close to uniform. Corrected item-total correlations ranged 0.796–0.872 (EE), 0.861–0.888 (DP), and 0.629–0.759 (PA), all far above the 0.30 criterion, and removing any single item never raised *α*. Values above 0.95 for EE and DP point to some item redundancy rather than a measurement problem (Tavakol and Dennick, 2011). Contrary to the expectation embedded in H4, PA reliability remained high, although its item-total correlations were the lowest of the three subscales. The weaker aspects of PA emerged instead in factor saturation, in the residual structure of the confirmatory model, and in its distinct pattern of external associations.

### 3.3 Exploratory factor analysis in the calibration half

Sampling adequacy in the calibration half was high (KMO = 0.942) and Bartlett’s test was significant (*χ*^2^(231) = 3430.21; *p* < 0.001). Three factors carried eigenvalues above 1.0 (10.811, 4.053, 1.748), accounting after extraction for 48.06, 16.71, and 7.03% of common variance (71.81% cumulatively). The rotated pattern matrix separated the items along the expected lines: the nine EE items loaded 0.756 to 0.920 on the first factor, the five DP items 0.794 to 0.906 on the third, and the eight PA items 0.688 to 0.807 on the second. One item reached the cross-loading threshold, PA1 loading 0.303 on the exhaustion factor. Factor correlations were 0.640 between EE and DP, −0.338 between EE and PA, and −0.257 between DP and PA. The complete pattern matrix and communalities appear in Supplementary Table S2.

Repeating the analysis in the full sample reproduced this solution (KMO = 0.952; Bartlett *χ*^2^(231) = 6643.63; *p* < 0.001; eigenvalues 10.680, 4.169, 1.616; 47.41, 17.24, and 6.38% of common variance, 71.03% cumulatively). All nine EE items loaded on the first factor (0.737 to 0.913), the five DP items on the third (0.800 to 0.905), and the eight PA items on the second (0.709 to 0.804), with no cross-loading above 0.30 (Tables 3, 4). Composite reliability was 0.959 (EE), 0.932 (DP), and 0.918 (PA), and AVE was 0.722, 0.733, and 0.583. Inter-factor correlations were 0.679, −0.331, and −0.290. HTMT ratios stayed below the 0.85 criterion (EE with DP 0.731; EE with PA 0.331; DP with PA 0.306), and the square roots of the AVE values obtained from the confirmatory model (0.862, 0.902, and 0.764) exceeded the largest latent correlation of 0.731, satisfying the Fornell-Larcker criterion.

**Table 3:** Total variance explained (principal axis factoring).

Factor	Initial eigenvalue	% of variance	Cumulative %	Extraction SS loadings	Extraction %	Extraction cumulative %
1	10.680	48.55	48.55	10.430	47.41	47.41
2	4.169	18.95	67.50	3.793	17.24	64.65
3	1.616	7.34	74.84	1.403	6.38	71.03

**Table 4:** Pattern matrix after Promax rotation (loadings ≥ 0.30 shown).

Item	Factor 1 (EE)	Factor 2 (PA)	Factor 3 (DP)
EE1	0.864		
EE2	0.887		
EE3	0.833		
EE4	0.913		
EE5	0.860		
EE6	0.867		
EE7	0.804		
EE8	0.868		
EE9	0.737		
DP1		0.882	
DP2		0.905	
DP3		0.800	
DP4		0.868	
DP5		0.821	
PA1			0.709
PA2			0.804
PA3			0.789
PA4			0.739
PA5			0.792
PA6			0.745
PA7			0.792
PA8			0.735

Extraction: principal axis factoring; rotation: Promax with Kaiser normalization; rotation converged in 5 iterations. Inter-factor correlations: EE-DP = 0.679; EE-PA = −0.331; DP-PA = −0.290.

### 3.4 Confirmatory factor analysis and comparison of competing models

In the validation half, the correlated three-factor model fitted well: *χ*^2^(206) = 347.6, *χ*^2^/df = 1.69; CFI = 0.955; TLI = 0.950; RMSEA = 0.065; SRMR = 0.080. Standardized loadings were high throughout (EE 0.819 to 0.897; DP 0.864 to 0.919; PA 0.679 to 0.814), and the latent correlations reproduced the exploratory picture (EE with DP 0.752; EE with PA − 0.316; DP with PA − 0.349). Internal consistency held in both halves (calibration α 0.965, 0.960, and 0.913; validation α 0.960, 0.950, and 0.922). Item-level loadings for the validation half appear in Supplementary Table S2.

Table 5 sets the three-factor model against three alternatives in both samples. The single-factor model failed decisively, and the two-factor model that merges exhaustion with depersonalization improved on it without approaching acceptable fit. The three-factor model outperformed both by a wide margin (full sample Δ*χ*^2^(3) = 2158.6 and Δ*χ*^2^(2) = 898.4; validation half Δ*χ*^2^(3) = 1050.3 and Δ*χ*^2^(2) = 380.0; all *p* < 0.001).

**Table 5:** Comparison of competing measurement models for the MBI-ES.

Model	*χ* ^2^	df	*χ*^2^/df	GFI	AGFI	NFI	TLI	CFI	RMSEA	SRMR	AIC	BIC
Full sample (*n* = 323)												
M1 One-factor	2633.44	209	12.60	0.421	0.299	0.614	0.593	0.632	0.190	0.186	2721.44	2887.66
M2 Two-factor (EE + DP/PA)	1373.26	208	6.60	0.628	0.548	0.799	0.803	0.823	0.132	0.091	1463.26	1633.26
M3 Three-factor correlated	474.88	206	2.31	0.884	0.857	0.930	0.954	0.959	0.064	0.067	568.88	746.43
M4 Bifactor	295.24	187	1.58	0.927	0.901	0.957	0.980	0.984	0.042	0.034	427.24	676.57
Validation subsample (*n* = 162)												
M1 One-factor	1397.89	209	6.69	0.408	0.284	0.593	0.589	0.628	0.188	0.194	1485.89	1621.75
M2 Two-factor (EE + DP / PA)	727.57	208	3.50	0.636	0.557	0.788	0.819	0.837	0.125	0.098	817.57	956.51
M3 Three-factor correlated	347.57	206	1.69	0.845	0.809	0.899	0.950	0.955	0.065	0.080	441.57	586.69
M4 Bifactor	249.16	187	1.33	0.884	0.843	0.927	0.976	0.980	0.045	0.050	381.16	584.94

Maximum likelihood estimation; *χ*^2^ computed on N − 1. Nested comparisons in the full sample: M1 versus M3, Δ*χ*^2^(3) = 2158.56; M2 versus M3, Δ*χ*^2^(2) = 898.38; M3 versus M4, Δ*χ*^2^(19) = 179.64; all *p* < 0.001. Validation subsample: Δ*χ*^2^(3) = 1050.32; Δ*χ*^2^(2) = 380.00; Δ*χ*^2^(19) = 98.41; all *p* < 0.001. The bifactor model was rejected on parameter interpretability despite superior global fit: hierarchical omega for the exhaustion-specific factor was 0.011 (validation half 0.005), six of its nine loadings fell below 0.10 and three were negative, and general-factor loadings on the eight PA items ranged from −0.41 to −0.07. Omega hierarchical for the general factor was 0.619, explained common variance 0.599, and the percentage of uncontaminated correlations 0.680. AIC and BIC follow the *χ*^2^ + 2*q* and *χ*^2^ + *q* ln(N) convention, with *q* = 44, 45, 47, and 66 free parameters. Full details of the bifactor solution appear in Supplementary Table S3.

The bifactor model returned better global indices than the three-factor model in both samples (full sample CFI = 0.984; RMSEA = 0.042; Δ*χ*^2^(19) = 179.6, *p* < 0.001), so we examined its parameters before drawing a conclusion. The solution proved uninterpretable. The exhaustion-specific factor collapsed: six of its nine standardized loadings fell below 0.10, three were negative, and its hierarchical omega reached only 0.011, leaving it with no reliable variance of its own. The general factor behaved as an exhaustion factor rather than a general burnout factor, loading 0.82 to 0.90 on the EE items but −0.41 to −0.07 on the eight PA items. Hierarchical omega for the general factor was 0.619, explained common variance 0.599, and the percentage of uncontaminated correlations 0.680. The same pattern appeared in the validation half, where the exhaustion-specific factor reached ω_HS = 0.005 and general-factor loadings on the PA items ran from −0.41 to −0.03 (Supplementary Table S3). A solution of this kind supports neither a single total burnout score nor any subscale score that the three-factor model does not already provide, and we retained the three-factor model on that basis.

In the full sample the three-factor model fitted as follows: *χ*^2^(206) = 474.9, *χ*^2^/df = 2.305; CFI = 0.959; TLI = 0.954; NFI = 0.930; RMSEA = 0.064 (90% CI 0.056 to 0.071); SRMR = 0.067. GFI (0.884) and AGFI (0.857) fell below their conventional thresholds. Both indices decline as the number of indicators grows and rise with the number of freely estimated parameters (Kenny and McCoach, 2003), so we neither discount the shortfall for the three-factor model nor read the higher values of the more heavily parameterized bifactor solution as evidence in its favour.

Local fit was good overall. The median absolute residual correlation was 0.032, and only 11 of 231 values exceeded 0.15. The residuals that did stand out clustered at the boundary between exhaustion and personal accomplishment and involved two items in particular. PA1 and PA2 carried positive residual correlations with most exhaustion items, the largest being EE7 with PA2 (0.205), which means that the model implies a stronger inverse relation to exhaustion than these items show. PA6 and PA8 ran in the opposite direction against EE6 (−0.188 and −0.156). Freeing the residual covariances of the two most content-redundant exhaustion pairs improved *χ*^2^ by 9.59 on two degrees of freedom (*p* = 0.008) and left SRMR unchanged, so item redundancy within the exhaustion block does not drive the residual structure. PA1 and PA2 also carried the lowest corrected item-total correlations of the subscale (0.629 and 0.695), and PA1 produced the single cross-loading observed in the calibration analysis. The three-factor model is thus an adequate representation with one identifiable weak point, and that weak point lies in the personal accomplishment subscale. Standardized loadings in the full sample were high and significant throughout (0.82 to 0.89 for EE, 0.88 to 0.91 for DP, 0.65 to 0.81 for PA), and latent correlations were 0.73, −0.36, and −0.32 (Figure 1).

**Figure 1:**
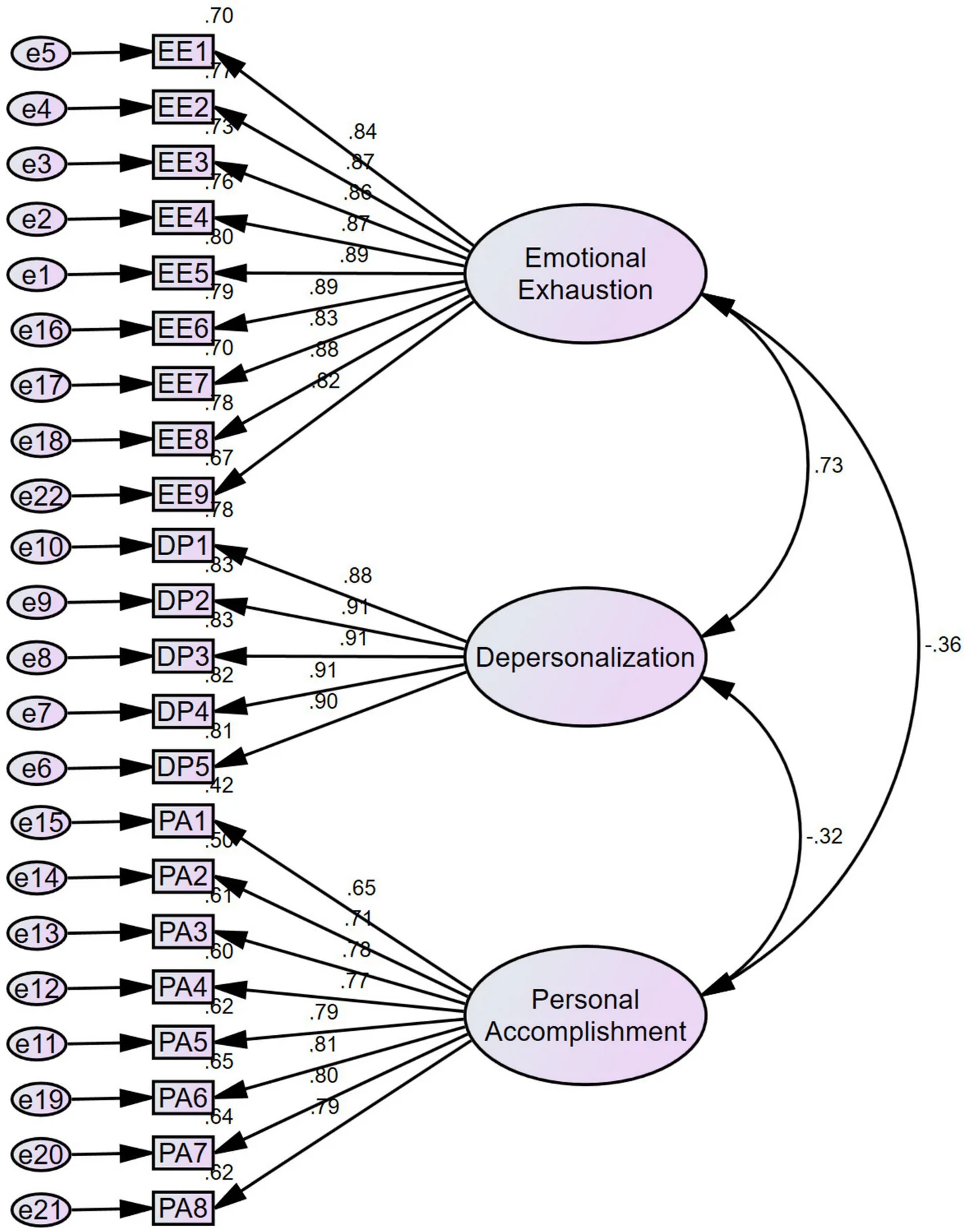
Confirmatory factor model of the three-factor MBI-ES structure in women teachers in Kazakhstan (*n* = 323). Latent factors (*EE*, emotional exhaustion; *DP*, depersonalization; *PA*, personal accomplishment) are shown with standardized loadings for each observed item; Double-headed arrows denote freely estimated latent correlations. Model fit: *χ*^2^(206) = 474.9, *χ*^2^/*df* = 2.305; *CFI* = 0.959; *TLI* = 0.954; *NFI* = 0.930; *RMSEA* = 0.064 (90% *CI* 0.056 to 0.071); *SRMR* = 0.067; *GFI* = 0.884; *AGFI* = 0.857.

#### 3.4.1 Measurement invariance

Across the two random halves the model held at every level: configural *χ*^2^(412) = 696.4, CFI = 0.957, RMSEA = 0.066; metric Δ*χ*^2^(19) = 12.8, *p* = 0.847, ΔCFI = +0.001; scalar Δ*χ*^2^(19) = 19.4, *p* = 0.428, ΔCFI = 0.000. Full scalar invariance thus supports the internal replicability of the measurement model.

Across the three study sites, configural (*χ*^2^(618) = 1007.2; CFI = 0.941; RMSEA = 0.077) and metric invariance held (Δ*χ*^2^(38) = 54.0, *p* = 0.045; ΔCFI = −0.002), so the items relate to the latent dimensions in the same way in each city. Scalar invariance did not hold (Δ*χ*^2^(38) = 109.4, *p* < 0.001; ΔCFI = −0.011). Item intercepts differ across sites, and the observed differences in mean EE and PA reported in Section 3.1 cannot be interpreted as differences in latent burnout level. Comparing Astana against the two other cities pooled, both metric and scalar invariance held (Δ*χ*^2^(19) = 25.7, *p* = 0.138; Δ*χ*^2^(19) = 22.4, *p* = 0.266), which places the intercept non-invariance in the contrasts between the two smaller sites. Full invariance results appear in Supplementary Table S4.

### 3.5 Bivariate associations

Table 6 reports Spearman correlations of all study variables with the three burnout dimensions. EE correlated most strongly with perceived work stress (ρ = 0.706), followed by state anxiety (ρ = 0.657), information overload (ρ = 0.485), emotional tension (ρ = 0.450), conflict frequency (ρ = 0.426), perceived social isolation (ρ = 0.385), and documentation load (ρ = 0.330); satisfaction with the interpersonal climate (ρ = −0.263), sleep duration (ρ = −0.261), and physical activity (ρ = −0.206) showed protective-direction associations. For DP, the strongest correlates were work stress (ρ = 0.580), state anxiety (ρ = 0.520), conflict frequency (ρ = 0.514), information overload (ρ = 0.422), and social isolation (ρ = 0.411), while interpersonal climate showed the strongest negative association (ρ = −0.353). PA related most strongly to state anxiety (ρ = −0.375) and, more modestly, to emotional tension (ρ = −0.232), a favorable interpersonal climate (ρ = 0.222), a favorable appraisal of digitalization (ρ = 0.219), work stress (ρ = −0.206), and social isolation (ρ = −0.164). Age (|ρ| ≤ 0.057), length of service, marital status, number of children, subject area, and weekly teaching hours showed no meaningful association with any dimension. The burnout dimensions intercorrelated as expected (EE-DP ρ = 0.722; EE-PA ρ = −0.332; DP–PA ρ = −0.335).

**Table 6:** Spearman rank correlations between predictor variables and burnout outcomes (*n* = 323).

Code	Variable	EE	DP	PA
X1	Age	−0.057	−0.043	0.024
X2	Length of service	−0.038	−0.034	0.015
X3	State anxiety (STAI-S)	0.657**	0.520**	−0.375**
X4	Marital status ᵃ	−0.076	−0.027	0.040
X5	Number of children	−0.044	0.029	0.031
X6	Subject area ᵃ	0.051	0.038	0.040
X7	Employment type	0.117*	0.071	0.072
X8	Weekly teaching hours	0.034	0.017	−0.038
X9	Total weekly workload	0.214**	0.181**	−0.065
X10	Administrative duties	0.227**	0.193**	0.098
X11	Documentation load	0.330**	0.278**	−0.018
X12	Conflict frequency	0.426**	0.514**	−0.091
X13	Interpersonal climate	−0.263**	−0.353**	0.222**
X14	Perceived social isolation	0.385**	0.411**	−0.164**
X15	Sleep duration	−0.261**	−0.176**	0.104
X16	Physical activity	−0.206**	−0.080	0.100
X17	Meal regularity	0.284**	0.191**	−0.115*
X18	Emotional tension	0.450**	0.358**	−0.232**
X19	Daily screen time	0.278**	0.235**	−0.008
X20	Information overload	0.485**	0.422**	−0.063
X21	Impact of digitalization	−0.157**	−0.136*	0.219**
X22	Perceived work stress	0.706**	0.580**	−0.206**

*p < 0.05; **p < 0.01 (two-tailed). ᵃNominal variables; coefficients are reported for completeness and interpreted only as absence of association.

### 3.6 Hierarchical models within the job demands-resources framework

Table 7 reports the variance each block added, and Table 8 the standardized coefficients before and after appraised strain entered. Collinearity stayed acceptable for the substantive predictors (VIF 1.03 to 2.31), age and length of service reached 6.45 and 6.14 because they correlate at r = 0.90, and a sensitivity model excluding length of service left every conclusion unchanged.

**Table 7:** Incremental variance explained by successive job demands-resources blocks (*n* = 323).

Step (variables entered)	EE Δ*R*^2^	EE *F* change (df)	DP Δ*R*^2^	DP *F* change (df)	PA Δ*R*^2^	PA *F* change (df)
1 Background characteristics (10)	0.048	1.58 (10, 312)	0.021	0.68 (10, 312)	0.034	1.08 (10, 312)
2 Job demands (8)	0.351	22.19 (8, 304)***	0.363	22.37 (8, 304)***	0.048	2.01 (8, 304)*
3 Job resources (5)	0.066	7.36 (5, 299)***	0.037	3.79 (5, 299)**	0.081	5.79 (5, 299)***
4 Appraised strain (3)	0.195	56.45 (3, 296)***	0.067	12.95 (3, 296)***	0.074	9.54 (3, 296)***
Total *R*^2^	0.660		0.488		0.237	
Adjusted *R*^2^	0.630		0.443		0.170	

EE, emotional exhaustion; DP, depersonalization; PA, personal accomplishment. Numbers in parentheses after each block give the count of variables entered. Block 1 comprised age, length of service, marital status, number of children, subject area, and employment type; block 2 weekly teaching hours, total weekly workload, administrative duties, documentation load, daily screen time, information overload, conflict frequency, and perceived social isolation; block 3 interpersonal climate, appraisal of digitalization, sleep duration, physical activity, and meal regularity; block 4 perceived work stress, emotional tension, and state anxiety. *p < 0.05; **p < 0.01; ***p < 0.001.

**Table 8:** Standardized regression coefficients before and after entry of appraised strain (*n* = 323).

Block	Predictor	EE step 3	EE final *β* [95% CI]	DP step 3	DP final *β* [95% CI]	PA step 3	PA final *β* [95% CI]
Job demands	Weekly teaching hours	0.006	0.001 [−0.09, 0.09]	−0.031	−0.031 [−0.14, 0.08]	−0.047	−0.040 [−0.17, 0.09]
	Total weekly workload	0.064	0.044 [−0.03, 0.12]	0.044	0.031 [−0.06, 0.12]	−0.092	−0.079 [−0.19, 0.03]
	Administrative duties	0.055	0.046 [−0.03, 0.12]	0.043	0.040 [−0.05, 0.13]	0.132*	0.140* [0.03, 0.25]
	Documentation load	0.044	0.026 [−0.06, 0.11]	0.029	0.013 [−0.09, 0.12]	0.043	0.062 [−0.06, 0.19]
	Daily screen time	−0.021	0.014 [−0.06, 0.09]	−0.051	−0.026 [−0.12, 0.07]	0.111	0.101 [−0.02, 0.22]
	Information overload	0.272***	0.067 [−0.03, 0.17]	0.177**	0.043 [−0.08, 0.16]	−0.015	0.045 [−0.10, 0.19]
	Conflict frequency	0.160**	0.062 [−0.02, 0.14]	0.330***	0.268*** [0.17, 0.37]	−0.040	0.002 [−0.12, 0.13]
	Perceived social isolation	0.180***	0.032 [−0.05, 0.11]	0.194***	0.117* [0.02, 0.22]	−0.070	0.005 [−0.12, 0.13]
Job resources	Interpersonal climate	−0.149**	−0.066 [−0.14, 0.01]	−0.180***	−0.131** [−0.22, −0.04]	0.171**	0.135* [0.03, 0.25]
	Appraisal of digitalization	−0.059	0.001 [−0.07, 0.07]	−0.026	0.006 [−0.08, 0.10]	0.185**	0.155** [0.04, 0.26]
	Sleep duration	−0.099*	−0.031 [−0.11, 0.04]	−0.024	0.012 [−0.08, 0.10]	0.136*	0.084 [−0.03, 0.19]
	Physical activity	−0.115*	−0.105** [−0.18, −0.03]	0.017	0.019 [−0.07, 0.11]	0.019	0.010 [−0.10, 0.12]
	Meal irregularity	0.121**	0.049 [−0.02, 0.12]	0.080	0.036 [−0.05, 0.13]	−0.076	−0.044 [−0.15, 0.07]
Appraised strain	Perceived work stress	—	0.320*** [0.22, 0.42]	—	0.239*** [0.11, 0.36]	—	0.039 [−0.11, 0.19]
	Emotional tension	—	0.087* [0.00, 0.17]	—	0.069 [−0.03, 0.17]	—	−0.128* [−0.25, 0.00]
	State anxiety (STAI-S)	—	0.323*** [0.23, 0.41]	—	0.126* [0.02, 0.23]	—	−0.291*** [−0.42, −0.16]

Step 3 gives standardized coefficients after background characteristics, job demands, and job resources had entered, before appraised strain. Final gives coefficients in the complete model. Background characteristics were retained in every model but produced no significant coefficient for any outcome and are reported in Supplementary Table S5, which also gives standard errors, confidence intervals for the step 3 estimates, and variance inflation factors. Variance inflation was 1.03 to 2.31 for all substantive predictors; age and length of service reached 6.45 and 6.14 owing to their correlation of *r* = 0.90, and their exclusion left all conclusions unchanged. *p < 0.05; **p < 0.01; ***p < 0.001.

For emotional exhaustion, background characteristics explained 4.8% of variance and did not reach significance as a block (*F*(10,312) = 1.58; *p* = 0.111). Job demands added 35.1% (*F*(8,304) = 22.19; *p* < 0.001) and job resources a further 6.6% (*F*(5,299) = 7.36; *p* < 0.001). At this point, before any strain appraisal entered, information overload (*β* = 0.272), perceived social isolation (*β* = 0.180), conflict frequency (*β* = 0.160), an unfavorable collegial climate (*β* = −0.149), irregular meals (*β* = 0.121), low physical activity (*β* = −0.115), and short sleep (*β* = −0.099) all contributed independently, and the model explained 46.5% of variance. Appraised strain added 19.5% (*F*(3,296) = 56.45; *p* < 0.001), bringing the model to *R*^2^ = 0.660 and adjusted *R*^2^ = 0.630. In the full model, state anxiety (*β* = 0.323), perceived work stress (*β* = 0.320), physical activity (*β* = −0.105), and emotional tension (*β* = 0.087) remained significant, while the demand and resource terms attenuated, indicating that the strain appraisals carry much of the variance previously attributable to demands.

For depersonalization, background characteristics contributed nothing (Δ*R*^2^ = 0.021; *p* = 0.739). Job demands added 36.3% (*F*(8,304) = 22.37; *p* < 0.001) and resources 3.7% (*F*(5,299) = 3.79; *p* = 0.002); conflict frequency (*β* = 0.330), perceived social isolation (*β* = 0.194), information overload (*β* = 0.177), and collegial climate (*β* = −0.180) were the active terms at that step. Appraised strain added 6.7% (*F*(3,296) = 12.95; *p* < 0.001), giving *R*^2^ = 0.488 and adjusted *R*^2^ = 0.443. Conflict frequency (*β* = 0.268), perceived work stress (*β* = 0.239), state anxiety (*β* = 0.126), social isolation (*β* = 0.117), and collegial climate (*β* = −0.131) all held in the full model, so the relational signature of depersonalization survives adjustment for appraised strain.

Personal accomplishment behaved differently. Background characteristics (Δ*R*^2^ = 0.034; *p* = 0.375) and job demands (Δ*R*^2^ = 0.048; *F*(8,304) = 2.01; *p* = 0.046) contributed little, whereas resources added 8.1% (*F*(5,299) = 5.79; *p* < 0.001), the largest block contribution for this outcome. At step 3 the active terms were a favorable appraisal of digitalization (*β* = 0.185), a favorable collegial climate (*β* = 0.171), longer sleep (*β* = 0.136), and administrative duties (*β* = 0.132). Appraised strain added 7.4% (*F*(3,296) = 9.54; *p* < 0.001), for *R*^2^ = 0.237 and adjusted *R*^2^ = 0.170. In the full model, state anxiety (*β* = −0.291) and emotional tension (*β* = −0.128) related negatively to accomplishment, while appraisal of digitalization (*β* = 0.155), administrative duties (*β* = 0.140), and collegial climate (*β* = 0.135) related positively. The complete coefficient set, including the background block and variance inflation factors, appears in Supplementary Table S5.

Three sensitivity analyses supported these models (Supplementary Table S6). Adjusting for study site left every substantive coefficient in place, with residual site effects for emotional exhaustion (Karaganda *β* = −0.283) and personal accomplishment (Karaganda *β* = 0.490; Petropavlovsk *β* = 0.629) but none for depersonalization. Removing length of service changed no conclusion (adjusted *R*^2^ 0.631, 0.445, and 0.168). Refitting the exhaustion model without state anxiety, whose content overlaps with the outcome, retained perceived work stress (*β* = 0.433), emotional tension (*β* = 0.123), physical activity (*β* = −0.119), social isolation (*β* = 0.106), and collegial climate (*β* = −0.083), with adjusted *R*^2^ = 0.566.

Model diagnostics supported the ordinary least squares assumptions (Figure 2). Histograms of standardized residuals approximated normality, P–P plots showed close agreement between observed and expected quantiles, and residual-versus-predicted scatterplots displayed no systematic heteroscedasticity or nonlinearity. The depersonalization panel shows a boundary along its lower left edge, which reflects the floor of the DP scale rather than model misspecification; 60 participants (18.6%) scored two or below, so residuals at low predicted values are bounded from beneath. Residual skewness stayed modest across the three models (0.04 for EE, 0.47 for DP, and −0.32 for PA), and no transformation was applied.

**Figure 2:**
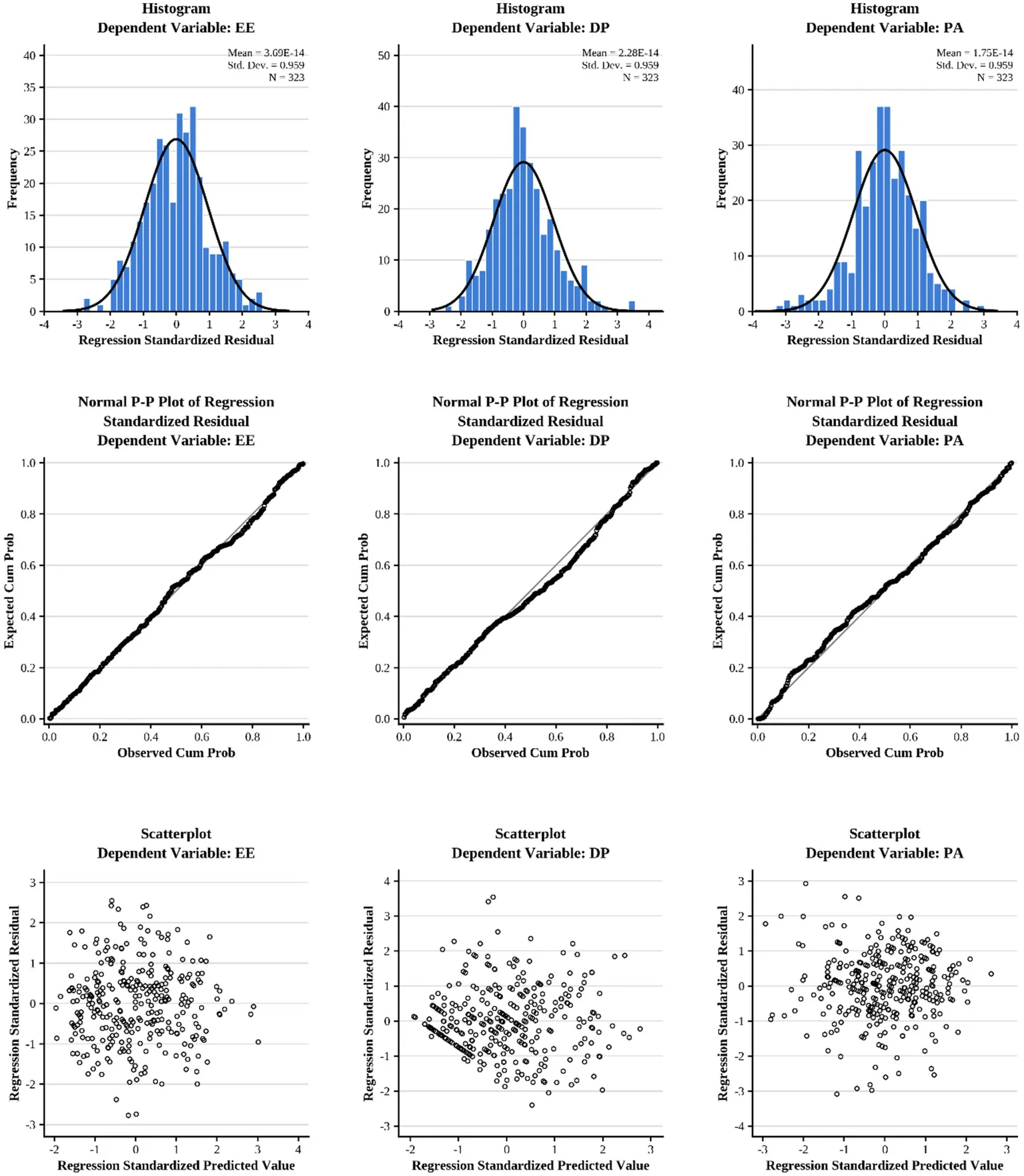
Diagnostic plots for the hierarchical regression models of *EE*, *DP*, and *PA*. Top: Histograms of standardized residuals with fitted normal curves; Middle: Normal P–P plots of standardized residuals; Bottom: Standardized residuals against standardized predicted values. Residuals approximate normality with no systematic heteroscedasticity (standardized residual ranges: *EE* −2.78 to 2.55; *DP* −2.40 to 3.54; *PA* −3.08 to 2.93; Durbin-Watson 1.827, 1.965, and 1.927).

#### 3.6.1 Exploratory stepwise models and their stability

For comparability with the wider burnout literature, Supplementary Table S7 reports stepwise models over the same variable pool. They retained seven predictors for emotional exhaustion, five for depersonalization, and five for personal accomplishment, reaching adjusted *R*^2^ values of 0.631, 0.463, and 0.173, close to those of the hierarchical models. Repeating the procedure in 500 bootstrap resamples shows that the retained sets are unequally stable (Supplementary Table S8). For emotional exhaustion, state anxiety and perceived work stress entered in 100% of resamples and physical activity in 86.4%, while information overload and emotional tension entered in 58.8% each, conflict frequency in 43.6%, and total weekly workload in 33.0%. For depersonalization, conflict frequency (100%), perceived work stress (99.0%), collegial climate (80.0%), perceived social isolation (68.0%), and state anxiety (66.4%) were comparatively stable. For personal accomplishment, state anxiety (99.6%), appraisal of digitalization (87.8%), and administrative duties (74.8%) were stable, while the STEM contrast (59.0%), collegial climate (57.8%), and emotional tension (53.2%) entered in roughly half of resamples. Median model size was 8 predictors for emotional exhaustion, 6 for depersonalization, and 7 for personal accomplishment, with ranges from 3 to 14. We restrict substantive interpretation to predictors that hold in both the hierarchical model and the resampling analysis.

#### 3.6.2 Common method variance

The first unrotated factor across all 36 self-report variables accounted for 36.6% of variance, with six factors above unity. A confirmatory single-factor model over the MBI items fitted poorly (*χ*^2^(209) = 2633.4; CFI = 0.632; TLI = 0.593; RMSEA = 0.190; SRMR = 0.186). Marker-variable adjustment using the smallest observed association between a questionnaire variable and a burnout score (ρ = 0.008) shifted the key correlations by no more than 0.005: work stress with EE from 0.706 to 0.703, conflict frequency with DP from 0.514 to 0.510, and collegial climate with DP from −0.353 to −0.348. Absolute correlations with the burnout dimensions ranged from 0.008 to 0.706 across variables collected in the same instrument at the same sitting, a spread inconsistent with uniform inflation by a single method factor.

## 4 Discussion

### 4.1 Principal findings

This study delivers two main results. First, the Russian-language MBI-ES reproduced its canonical three-factor structure in women schoolteachers in Kazakhstan, and it did so under a design that separates exploration from confirmation and that pits the canonical model against plausible competitors. The structure emerged in a calibration half, held in an untouched validation half, and outperformed both a single-factor and a merged exhaustion-cynicism model by a wide margin, extending the instrument’s cross-cultural evidence base (Worley et al., 2008; Aguayo et al., 2011) to a Central Asian population for which such data were absent. Given that the oblique three-factor solution has failed in several sizable recent samples in favor of bifactor, two-factor, or single-factor alternatives (Hawrot and Koniewski, 2018; De Beer and Bianchi, 2019; Lemos et al., 2025), the clean separation obtained here is not a foregone conclusion. A bifactor model did return better global fit indices, as it commonly does, but its exhaustion-specific factor carried no reliable variance and its general factor loaded negatively on the accomplishment items, so it identifies no score that the three-factor model does not already provide. Loadings and intercepts proved invariant across the two random halves, and loadings held across the three study sites. Item intercepts did not, and the interpretive consequence deserves stating in full. Teachers in different cities who stand at the same latent level do not produce the same expected item responses, so the site differences in observed exhaustion and accomplishment reported in Section 3.1 are differences in observed scores and cannot be read as evidence that burnout is higher in Astana than in Karaganda. The residual site coefficients that survived adjustment in the regression models rest on the same observed sums and carry the same qualification. Contrasting Astana against the two other cities pooled, both metric and scalar invariance held, so a latent comparison in that two-group configuration would be defensible; we did not estimate latent means and make no claim about their ordering. The intercept differences lie in the contrast between the two smaller sites. Any use of this instrument for cross-site occupational health monitoring should establish invariance before levels are compared, while within a single city the three subscale scores remain usable. The same caution governs comparison with published samples from other countries, since invariance of the Russian-language MBI-ES against other language versions has not been tested and recent multi-country work with this instrument has reached only partial scalar invariance (Aboagye et al., 2018; Lemos et al., 2025). Second, the pattern of correlates followed the JD-R logic closely. EE, the depletion pathway, was tied above all to perceived work stress and state anxiety, with information overload, conflict frequency, and perceived social isolation contributing before those appraisals entered the model, and it reached the highest explanatory power of the three outcomes. DP, the withdrawal pathway, carried a distinctly relational signature, combining work stress and anxiety with conflict frequency and social isolation, and receiving its only protective contribution from a favorable interpersonal climate. PA stood apart, with a mixed profile of psychoemotional and resource correlates and the lowest explained variance. Regular physical activity remained an independent protective correlate of emotional exhaustion after adjustment, and a favorable interpersonal climate of depersonalization, while sleep duration lost its independent contribution once appraised strain entered.

The primacy of subjective appraisals over formal job parameters deserves emphasis, and the size of the gap is easier to judge against published estimates. Meta-analytic work on the MBI has repeatedly placed demand-type correlates such as role conflict and workload at or above 0.50 with emotional exhaustion, and has found demand correlates more strongly related to exhaustion than resource correlates are (Lee and Ashforth, 1996; Alarcon, 2011). Perceived work stress in our sample sat above that band (ρ = 0.706), while the two quantitative workload indicators fell far below it (weekly teaching hours ρ = 0.034; total weekly working time ρ = 0.214). The contrast in our data is not between demands and exhaustion but between demands counted in hours and demands registered as strain. Age, length of service, marital status, number of children, subject area, employment type, and contracted teaching hours were at most weakly related to any burnout dimension. No formal workload indicator retained an independent contribution to exhaustion once appraised strain entered the model, and administrative responsibilities related positively to personal accomplishment rather than to either strain dimension. This ordering mirrors longitudinal Norwegian evidence that perceived time pressure and workload track emotional exhaustion more closely than formal characteristics do (Skaalvik and Skaalvik, 2020), and it matches the determinants that survive prospective designs, since a systematic review of 33 longitudinal studies identified job satisfaction, work climate and pressure, teacher self-efficacy, neuroticism, perceived collective exhaustion, and classroom disruption as detrimental determinants of teacher exhaustion, none of them contractual (Mijakoski et al., 2022). TALIS data point the same way: administrative tasks occupy a small share of teachers’ working time yet rank among the most frequently named sources of stress, and reported stress tracks the experience of continuous and unsupported change more closely than the volume of work (OECD, 2025). The pattern is consistent with the JD-R proposition that wellbeing follows the subjective balance of demands and resources (Bakker et al., 2023).

The anxiety finding sits at the upper end of its published range rather than inside it. A systematic review and meta-analysis put the pooled burnout-anxiety correlation at 0.460 (95% CI 0.421 to 0.497) and reported smaller effects in studies using the MBI, against ρ = 0.657 between state anxiety and exhaustion here (Koutsimani et al., 2019). Two features of the design plausibly inflate our estimate: the STAI-S was administered at the same sitting as the MBI-ES, and its content lies close to the exhaustion items. Given the cross-sectional design and that conceptual proximity, the association should be read strictly as concurrent, with no direction implied, which is why these variables enter the hierarchical models last. A sensitivity model that removed state anxiety left work stress, physical activity, and the remaining predictors significant (adjusted *R*^2^ = 0.566), so the exhaustion findings do not rest on the anxiety term. A recent Canadian study of 780 teachers assessed with the same instrument reproduced the gradient we observed across dimensions rather than its absolute size, with teachers reporting high perceived stress 6.9 times more likely to show high emotional exhaustion, 2.6 times more likely to show high depersonalization, and 2.3 times more likely to report low accomplishment (Agyapong et al., 2024). Our rank correlations descend in the same order (0.706, 0.580, and −0.206), which suggests that appraised strain distributes across the three dimensions in a comparable way in two very different school systems. The broader link between anxiety and teacher burnout is documented across countries (Agyapong et al., 2022; Westphal et al., 2022; Hooker et al., 2025), while the same meta-analysis treats burnout and anxiety as related yet distinct constructs.

The relational profile of depersonalization replicates the classic conflict-cynicism route (Leiter and Maslach, 2004) and the resource-conservation account of isolation (Halbesleben, 2006), and the ordering within it departs from what the rest of the model shows. Every appraised-strain variable correlated more strongly with exhaustion than with depersonalization, as the wider literature would predict, yet interpersonal conflict reversed that order (ρ = 0.514 against 0.426) and perceived isolation reversed it marginally (0.411 against 0.385). Conflict then held its position in the adjusted model after every quantitative demand had dropped out. Relational strain in our data acted more directly on the appraisal of other people than on the energy budget, which is what the withdrawal pathway anticipates. Within the relational block the demand side outweighed the resource side, with isolation at 0.411 against collegial climate at −0.353, in line with the general finding that demands relate to burnout more strongly than resources do (Lee and Ashforth, 1996; Alarcon, 2011). The protective climate effect follows the evidence that collegial support and a positive psychological climate are among the more stable correlates of lower burnout, with feeling marginalized by coworkers recurring as a burnout factor (Nwoko et al., 2023; Sohail et al., 2023; Gao and Wang, 2026). One departure from prior evidence should be recorded rather than smoothed over. Halbesleben (2006) reported that work-based sources of support relate more closely to exhaustion than to the other two dimensions, whereas satisfaction with the collegial climate here retained an independent association with depersonalization and with accomplishment but not with exhaustion. A resource that works through belonging and recognition rather than through workload relief may attach to the withdrawal and efficacy pathways rather than to the energy pathway. We hold that reading loosely, since the review of longitudinal studies found protective determinants to be the least consistent group across waves and samples (Mijakoski et al., 2022).

The retention of physical activity in the exhaustion model aligns with the recovery literature, in which activity supports psychological detachment from work demands and buffers strain (Sonnentag and Fritz, 2015; Xu et al., 2025). Evidence from within the JD-R framework points the same way: moderate-intensity activity relates to lower burnout, and vigorous activity can buffer the demands-strain relationship (Fodor et al., 2020). The finding identifies a modifiable individual-level resource alongside the organizational ones. The documentation and information-overload findings, together with the sizable bivariate correlations of screen time with EE and DP, situate the results within the technostress literature, in which digital communication pressure itself predicts teacher burnout (Tarafdar et al., 2015; Califf and Brooks, 2020; Qin et al., 2025), and within the specific Kazakhstani context of rapid curricular reform and digitalization (Karabassova, 2020; Nurmukhamed et al., 2025).

Personal accomplishment followed a different logic from the strain pathway. Beyond the negative contributions of state anxiety and emotional tension, accomplishment was higher among teachers reporting a heavier load of administrative duties and among those who appraised digitalization as simplifying their work. These positive associations are consistent with resource-based accounts in which role breadth and technology experienced as an aid, rather than a demand, feed professional self-efficacy (Skaalvik and Skaalvik, 2018; Fernández-Batanero et al., 2021; Li, 2023; Uslukaya, 2024). The cross-sectional design leaves the direction open: more accomplished teachers may also be the ones entrusted with administrative roles and the ones who master digital tools. Personal resources such as self-efficacy, optimism, and resilience, which relate to lower burnout directly and through perceived social support (Dicke et al., 2018; Sohail et al., 2023; Uslukaya, 2024), were not measured and likely account for part of the unexplained variance.

Some of these results are harder to detach from their setting than others. The demands that carried most weight are largely the ones the recent reform cycle produced. Documentation load correlated with exhaustion at 0.330 and information overload at 0.485, and 65.6% of participants carried duties beyond the classroom, in a system that between 2012 and 2025 introduced an updated curriculum with expanded formative assessment and reporting, a trilingual education policy, and a rapid rollout of digital platforms into a workforce whose digital pedagogical preparation was uneven (Karabassova, 2020; OECD, 2023). Reform-related stress already predicts turnover intentions among Kazakhstani teachers in TALIS data (Nurmukhamed et al., 2025). Greater self-rated administrative involvement related to higher personal accomplishment without relating independently to either strain dimension, which runs against the assignment of this variable to the demands block. The item recorded the amount of administrative work on a three-point scale and captured nothing about the form these duties took, so we can say that more administrative involvement accompanied a stronger sense of professional effectiveness, and no more than that. Broader role content is one reading. The design cannot separate it from the possibility that teachers with a stronger sense of efficacy are the ones given more of this work, or that the same disposition shapes both ratings. The appraisal of digitalization as simplifying work, endorsed by 57.3% of participants and independently related to accomplishment, is likewise tied to the platforms and support arrangements of this system rather than to digitalization as a general condition. One discrepancy with national survey evidence needs stating plainly. TALIS 2024 places Kazakhstan among the education systems reporting the least teacher stress, with fewer than 5% of teachers reporting a great deal of stress against an average of 19% across participating systems, and around half reporting none at all (OECD, 2025). Our sample reported modest formal hours, with only 8.7% above 40 total weekly hours, yet 31.0% reported work stress arising often or always and 20.4% reached the high band of state anxiety. Item wording, response format, and mode of administration all plausibly contribute to the gap. Our questionnaires were anonymous, carried no school identifier, and were returned independently of school management, which removes one source of pressure toward favourable answers. The two sources measure different things and are not in direct conflict, but the size of the difference argues against relying on single-item national stress indicators when occupational health monitoring is planned for this workforce.

### 4.2 Gender, vulnerability, and wellbeing in a feminized profession

Because women constitute 82.1% of Kazakhstan’s teaching workforce, the occupational conditions documented here are, in practice, the working conditions of a predominantly female labor force, and burnout in this setting functions as an indicator of gendered occupational vulnerability as much as an individual outcome. Women teachers report higher emotional exhaustion than men in meta-analytic syntheses (Purvanova and Muros, 2010; Agyapong et al., 2022). The same asymmetry appears in recent primary data. In a Canadian sample assessed with the MBI-ES, male teachers were about 2.4 times as likely as their female colleagues to show high depersonalization (Agyapong et al., 2024). Our design cannot test that contrast, and we report it only to locate this sample within the wider pattern. Cohort evidence shows that they combine heavier emotional and quantitative job demands with a larger unpaid domestic load (Stengård et al., 2022), a combination that leaves little room for recovery. Our finding that burnout is associated with perceived psychosocial load rather than with contracted hours is consistent with that picture: the strain of this workforce is concentrated in emotional labor, relational friction, and information pressure, dimensions that formal workload accounting does not capture. For a sample in which roughly two thirds are married and more than three quarters have children, the interplay between occupational and domestic demands is plausibly part of the unmeasured background of the stress and anxiety appraisals observed here. Framing teacher burnout as a wellbeing and equity problem of a feminized profession, rather than a matter of individual coping alone, argues for organizational-level responses and adds Central Asian evidence to a literature still dominated by Western samples.

Two consequences of studying women only should stay in view when these results are read. The design estimates associations inside a female workforce and offers no basis for comparing women with men, since the 36 men who completed the questionnaire were concentrated in physical education, technology, and pre-conscription training and could not have formed a comparable group. The gender differences reported in meta-analytic syntheses are background to this study rather than something it tests. The associations reported here may also differ in mixed-gender or male-dominated teaching populations, in which the balance of emotional labor, domestic load, and relational strain is likely to shift. What the design does secure is internal homogeneity: by removing gender and its confounding with subject specialization from the variable set, it isolates the psychosocial correlates of burnout within the group that constitutes 82.1% of the national teaching workforce, and it describes the conditions under which most Kazakhstani teachers actually work.

### 4.3 Implications for occupational health practice

Before practical directions are drawn, it helps to separate what belongs to this setting from what is likely to travel. The three-factor structure of the MBI-ES, the organization of correlates around an energy pathway and a withdrawal pathway, the precedence of appraised over counted demands, the relational signature of depersonalization, and the protective association of regular physical activity with exhaustion all reproduce patterns established in European, North American, and East Asian samples (Alarcon, 2011; Skaalvik and Skaalvik, 2020; Mijakoski et al., 2022; Nwoko et al., 2023; Bakker et al., 2023; Xu et al., 2025). Their appearance in a Central Asian, Russian-language, post-Soviet setting adds a data point to that literature rather than an exception to it, which is informative for an evidence base built largely on Western samples. Transfer is most plausible to school systems that share the governance features of this one: centrally set curricula and reporting requirements, workload defined in contractual teaching hours, a strongly feminized workforce, and a recent history of rapid digitalization. Other post-Soviet and Central Asian systems fit that description most closely, and the regional literature points in compatible directions (Kochetkov et al., 2023; Tokarskaya et al., 2025; Rezvanova, 2026). Systems in which schools hold substantial discretion over workload, staffing, and reporting may show a different balance, since the demands that dominate here are set largely above the level of the school. Two elements should not be carried across at all: the observed subscale means, for the invariance reasons, and the ordering of the three cities, for the same reason.

Three practical directions follow, all conditional on the associational nature of the evidence. First, because subjectively perceived stressors outweighed formal job parameters, prevention programs should reach beyond hour counts toward the organizational environment: reducing avoidable documentation and reporting duplication, managing conflict constructively, strengthening collegial support, and cultivating a supportive school digital culture, which has been shown to mitigate techno-stressors in K-12 settings (Chen et al., 2026). These directions are consistent with JD-R-based work identifying feedback, supervisor support, and role clarity as protective resources (Skaalvik and Skaalvik, 2018; Rezvanova, 2026) and with regional analyses of teacher burnout factors (Kochetkov et al., 2023). Second, the consistent association of state anxiety with all three dimensions supports including brief psychoemotional screening in routine occupational health monitoring of school staff, aimed not at profiling individuals but at the timely identification of teachers under marked strain who need organizational or psychological support. Third, the models point to inexpensive components of school wellbeing policy at two levels: team-level measures, from conflict-resolution procedures to structured peer support, and individual-level promotion of recovery through regular physical activity. Intervention evidence gives these directions a footing: a meta-analysis of 26 studies, mostly randomized trials, found a pooled reduction in schoolteachers’ stress outcomes, strongest for cognitive-behavioral programs, with moderate-quality evidence (Paudel et al., 2022), and interventions have achieved small to large reductions in depression and anxiety symptoms in teacher samples (Hooker et al., 2025). Combined programs that pair organizational change with the development of individual resources appear the most promising configuration in current reviews (Nwoko et al., 2023). The cross-sectional design of the present study still permits none of its own directions to be treated as demonstrated interventions. Therefore, they are hypotheses for prospective and interventional evaluation.

### 4.4 Limitations

Several limitations qualify the results. The cross-sectional design supports no inference about direction or causality, and reverse or reciprocal influence between anxiety, stress appraisals, and burnout cannot be excluded. Longitudinal evidence shows that determinants of teacher burnout separate reliably only in prospective designs (Mijakoski et al., 2022).

All data are self-reported and share method variance. Three checks argue against a dominant method factor: the first unrotated factor across all self-report variables reached 36.6%, a confirmatory single-factor model fitted poorly, and marker-variable adjustment shifted the key correlations by no more than 0.005. None of these eliminates the concern. We considered an unmeasured latent method construct but do not report one, because in a single-instrument design whose exhaustion and cynicism factors correlate at 0.73, an added method factor cannot be distinguished from a general burnout factor. Our estimation confirmed that the two solutions were numerically almost identical. The conceptual proximity of state anxiety, perceived work stress, and emotional tension to emotional exhaustion requires caution, which is why these variables enter the hierarchical models last and why we report an exhaustion model estimated without state anxiety. Personal resources known to shape personal accomplishment, such as self-efficacy and resilience, were not measured and probably account for part of the unexplained variance in that model. The conflict item recorded frequency without identifying the counterpart, so the relational signature of depersonalization cannot be attributed to conflict with colleagues rather than with parents, students, or the administration, and the practical implications we draw are correspondingly general. On the measurement side, the three-factor model is an adequate rather than an exact representation. Residual correlations concentrate at the boundary between exhaustion and personal accomplishment and involve two accomplishment items whose item-total correlations are the lowest in the subscale, so the interpretive caution that applies to that subscale in cross-cultural work applies here as well. Intercept non-invariance across the three study sites means that mean burnout levels are not directly comparable between cities, and any use of the instrument for cross-site monitoring should establish invariance first.

Four features of the design bound the population to which these estimates apply, and they should frame any transfer of the findings. Recruitment proceeded by convenience through school administrations in three cities, so the sample is not probabilistically representative of Kazakhstani teachers, and teachers absent on the survey day, including any absent through illness or exhaustion, had no chance of selection. The study covers women only, estimates associations inside a female workforce, and supports no conclusion about gender differences. Every participating school was an urban state general education school, which leaves rural teachers and the distinct conditions of small rural schools outside the frame. And because the questionnaire carried no school identifier, we can neither quantify clustering within schools nor fit a school-level random effect. Teachers in the same school share a principal, a workload allocation, a collegial climate, and a documentation regime, so the effective sample size is smaller than 323, the standard errors we report are probably too small, and the precision of the organizational estimates, collegial climate above all, is overstated to a degree we cannot measure. Nothing in the analysis separates variance belonging to schools from variance belonging to individuals. The stepwise models reported for comparability are exploratory, and the bootstrap analysis shows several of their weaker terms entering in fewer than half of resamples, so those coefficients require confirmation in independent samples.

## 5 Conclusion

The Russian-language MBI-ES reproduced its three-factor structure with high reliability and adequate validity in women schoolteachers in Kazakhstan, held under split-sample cross-validation, and outperformed single-factor and merged exhaustion-cynicism alternatives. The structure is suitable for occupational health monitoring in this population, with personal accomplishment read as a partially independent dimension and with mean comparisons across sites made only after invariance has been established. Within the job demands-resources framework, job demands and job resources together explained 46.5% of the variance in emotional exhaustion and 42.1% in depersonalization before any strain appraisal entered the models, and interpersonal conflict, perceived social isolation, information overload, and an unfavorable collegial climate carried most of that contribution. Appraised strain in the form of perceived work stress, state anxiety, and emotional tension added substantially to the exhaustion model. Regular physical activity was associated with lower exhaustion and a favorable collegial climate with lower depersonalization, while demographic and formal workload characteristics contributed little.

These are associations observed at a single point in time in a convenience sample of women teachers from urban schools in three cities, analysed without any account of clustering within schools, and they establish neither direction nor cause. Read with that constraint, they place teacher burnout in this feminized workforce closer to psychosocial working conditions than to contractual load, and they support the case for evaluating psychoemotional monitoring, improvement of the school social environment, and recovery-promoting measures as candidate components of programs protecting the professional health of educators in Central Asia. Prospective and interventional studies are needed to establish temporal ordering and to test whether the directions proposed here produce benefit.

## Data Availability

The raw data supporting the conclusions of this article will be made available by the authors, without undue reservation.
