# Supplementary material for: Occupational burnout in women schoolteachers in Kazakhstan: psychometric evaluation and psychosocial correlates within the job demands-resources framework
Source: Front Psychol. 2026 Sep 29;17:1945234. doi: 10.3389/fpsyg.2026.1945234 (PMC13658548; doi:10.3389/fpsyg.2026.1945234)
Supplement: Supplementary file 1 [file Supplementary_file_1.docx]

Supplementary Material

# 1 Supplementary Tables

**Supplementary Table S1** Codebook of the study variables (N = 323).

| **Code** | **Variable** | **Response categories** | **Domain** |
| --- | --- | --- | --- |
| **X1** | Age, years | continuous | Sociodemographic |
| **X2** | Length of service, years | continuous | Sociodemographic |
| **X3** | State anxiety, STAI-S | raw score, 20-80 | Psychoemotional |
| **X4** | Marital status | 1 single; 2 married; 3 divorced; 4 widowed | Sociodemographic |
| **X5** | Number of children | 1 none; 2 one; 3 two; 4 three or more | Sociodemographic |
| **X6** | Subject area | 1 primary; 2 STEM; 3 languages/humanities; 4 arts/PE/technology | Occupational |
| **X7** | Employment type | 1 part-time; 2 full-time; 3 above full contract | Occupational |
| **X8** | Weekly teaching hours | 1 <18; 2 18-24; 3 25-30; 4 >30 | Workload |
| **X9** | Total weekly working hours | 1 <30; 2 30-40; 3 40-50; 4 >50 | Workload |
| **X10** | Administrative duties | 1 none; 2 moderate; 3 substantial | Workload |
| **X11** | Documentation and reporting load | 1 very low to 5 excessive | Workload |
| **X12** | Frequency of interpersonal conflicts | 1 none; 2 1-2/yr; 3 3-4/yr; 4 >4/yr | Social-psychological |
| **X13** | Satisfaction with interpersonal climate | 1 very low to 5 very high | Social-psychological |
| **X14** | Perceived social isolation | 1 never to 5 always | Social-psychological |
| **X15** | Sleep duration | 1 <5 h; 2 5-6 h; 3 7-8 h; 4 >8 h | Lifestyle |
| **X16** | Physical activity frequency | 1 none to 4 ≥4/wk | Lifestyle |
| **X17** | Meal regularity | 1 regular; 2 irregular; 3 often skipped | Lifestyle |
| **X18** | Subjective emotional tension at work | 1 very low to 5 very high | Psychoemotional |
| **X19** | Daily screen time | 1 <2 h; 2 2-4 h; 3 4-6 h; 4 >6 h | Digital-informational |
| **X20** | Perceived information overload | 1 never to 4 always | Digital-informational |
| **X21** | Perceived impact of digitalization | 1 complicates greatly to 5 simplifies greatly | Digital-informational |
| **X22** | Perceived work stress | 1 none; 2 rarely; 3 often; 4 always | Psychoemotional |

**Supplementary Table S2** Split-sample cross-validation of the MBI-ES factor structure.

**Panel A. Pattern matrix, calibration half (principal axis factoring, Promax rotation, n = 161)**

| **Item** | **EE** | **PA** | **DP** | **h²** |
| --- | --- | --- | --- | --- |
| EE1 | **0.892** | 0.061 | 0.023 | 0.737 |
| EE2 | **0.920** | 0.054 | 0.007 | 0.807 |
| EE3 | **0.830** | 0.037 | 0.079 | 0.758 |
| EE4 | **0.881** | 0.041 | 0.004 | 0.748 |
| EE5 | **0.838** | 0.095 | 0.032 | 0.802 |
| EE6 | **0.895** | 0.082 | 0.011 | 0.845 |
| EE7 | **0.757** | 0.013 | 0.119 | 0.697 |
| EE8 | **0.874** | 0.049 | 0.009 | 0.806 |
| EE9 | **0.756** | 0.018 | 0.094 | 0.661 |
| DP1 | 0.001 | 0.022 | **0.895** | 0.811 |
| DP2 | 0.055 | 0.017 | **0.906** | 0.880 |
| DP3 | 0.200 | 0.042 | **0.794** | 0.854 |
| DP4 | 0.020 | 0.007 | **0.890** | 0.818 |
| DP5 | 0.147 | 0.034 | **0.794** | 0.820 |
| PA1 | 0.303 | **0.705** | 0.142 | 0.461 |
| PA2 | 0.161 | **0.787** | 0.013 | 0.557 |
| PA3 | 0.016 | **0.807** | 0.027 | 0.632 |
| PA4 | 0.253 | **0.688** | 0.127 | 0.586 |
| PA5 | 0.013 | **0.769** | 0.052 | 0.622 |
| PA6 | 0.110 | **0.745** | 0.054 | 0.654 |
| PA7 | 0.179 | **0.756** | 0.127 | 0.634 |
| PA8 | 0.083 | **0.744** | 0.015 | 0.609 |

*Sampling adequacy and extraction.* KMO = 0.942; Bartlett χ²(231) = 3430.21, p < 0.001. Eigenvalues 10.811, 4.053, and 1.748, with the fourth at 0.616. Extraction sums of squared loadings 10.574, 3.676, and 1.548, accounting for 48.06%, 16.71%, and 7.03% of common variance (71.81% cumulatively). Factor correlations: EE with DP 0.640; EE with PA −0.338; DP with PA −0.257.

**Panel B. Confirmatory standardized loadings, validation half (n = 162)**

| **Item** | **Factor** | **λ** | **R²** | **Item** | **Factor** | **λ** | **R²** |
| --- | --- | --- | --- | --- | --- | --- | --- |
| EE1 | EE | 0.819 | 0.671 | DP2 | DP | 0.881 | 0.776 |
| EE2 | EE | 0.853 | 0.728 | DP3 | DP | 0.899 | 0.808 |
| EE3 | EE | 0.842 | 0.710 | DP4 | DP | 0.919 | 0.845 |
| EE4 | EE | 0.882 | 0.778 | DP5 | DP | 0.891 | 0.793 |
| EE5 | EE | 0.897 | 0.804 | PA1 | PA | 0.679 | 0.462 |
| EE6 | EE | 0.859 | 0.738 | PA2 | PA | 0.715 | 0.512 |
| EE7 | EE | 0.826 | 0.682 | PA3 | PA | 0.774 | 0.599 |
| EE8 | EE | 0.877 | 0.769 | PA4 | PA | 0.808 | 0.652 |
| EE9 | EE | 0.824 | 0.679 | PA5 | PA | 0.786 | 0.618 |
| DP1 | DP | 0.864 | 0.746 | PA6 | PA | 0.814 | 0.663 |
|  |  |  |  | PA7 | PA | 0.806 | 0.649 |
|  |  |  |  | PA8 | PA | 0.792 | 0.628 |

*Fit and latent correlations.* χ²(206) = 347.57, χ²/df = 1.69; CFI = 0.955; TLI = 0.950; RMSEA = 0.065; SRMR = 0.080. EE with DP 0.752; EE with PA −0.316; DP with PA −0.349.

**Panel C. Internal consistency in each half**

| **Subscale** | **Calibration α (n = 161)** | **Validation α (n = 162)** |
| --- | --- | --- |
| Emotional exhaustion | 0.965 | 0.960 |
| Depersonalization | 0.960 | 0.950 |
| Personal accomplishment | 0.913 | 0.922 |

The sample was split at random with a fixed seed, stratified by study site. Loadings in Panel A are shown as absolute values with the highest in bold; the raw solution carried a common sign reversal on all three factors, which is arbitrary. Only one item reached the 0.30 cross-loading criterion (PA1 on the exhaustion factor, 0.303). Panel B rests on the untouched validation half.

**Supplementary Table S3** Bifactor solution: standardized loadings and reliability indices

| **Item** | **General** | **Specific EE** | **Specific DP** | **Specific PA** |
| --- | --- | --- | --- | --- |
| EE1 | 0.816 | 0.307 |  |  |
| EE2 | 0.853 | 0.387 |  |  |
| EE3 | 0.838 | 0.231 |  |  |
| EE4 | 0.870 | 0.038 |  |  |
| EE5 | 0.886 | 0.089 |  |  |
| EE6 | 0.892 | −0.007 |  |  |
| EE7 | 0.839 | −0.014 |  |  |
| EE8 | 0.903 | −0.093 |  |  |
| EE9 | 0.834 | −0.105 |  |  |
| DP1 | 0.617 |  | 0.636 |  |
| DP2 | 0.643 |  | 0.651 |  |
| DP3 | 0.705 |  | 0.578 |  |
| DP4 | 0.657 |  | 0.623 |  |
| DP5 | 0.675 |  | 0.590 |  |
| PA1 | −0.078 |  |  | 0.675 |
| PA2 | −0.068 |  |  | 0.753 |
| PA3 | −0.257 |  |  | 0.739 |
| PA4 | −0.365 |  |  | 0.686 |
| PA5 | −0.229 |  |  | 0.757 |
| PA6 | −0.413 |  |  | 0.702 |
| PA7 | −0.298 |  |  | 0.741 |
| PA8 | −0.378 |  |  | 0.693 |

**Bifactor reliability indices**

| **Index** | **General** | **Specific EE** | **Specific DP** | **Specific PA** |
| --- | --- | --- | --- | --- |
| Omega hierarchical (full sample) | 0.619 | 0.011 | 0.445 | 0.814 |
| Omega hierarchical (validation half) | — | 0.005 | 0.412 | 0.842 |
| Explained common variance (full sample) | 0.599 | 0.021 | 0.120 | 0.261 |
| Explained common variance (validation half) | 0.593 | — | — | — |

Omega total 0.952; percentage of uncontaminated correlations 0.680. Six of the nine exhaustion-specific loadings fall below 0.10 and three are negative, and the general factor loads negatively on all eight personal accomplishment items. The exhaustion-specific factor therefore retains no reliable variance of its own, and the general factor does not represent a general burnout dimension. The same pattern appears in the validation half. This solution was rejected in favour of the correlated three-factor model despite its superior global fit indices (Table 5).

**Supplementary Table S4** Measurement invariance of the three-factor MBI-ES model

| **Grouping** | **Model** | **χ²** | **df** | **CFI** | **TLI** | **RMSEA** | **Δχ²** | **Δdf** | **p** | **ΔCFI** | **ΔRMSEA** |
| --- | --- | --- | --- | --- | --- | --- | --- | --- | --- | --- | --- |
| Calibration vs validation half | Configural | 696.37 | 412 | 0.957 | 0.952 | 0.066 |  |  |  |  |  |
|  | Metric | 709.21 | 431 | 0.958 | 0.955 | 0.063 | 12.84 | 19 | 0.847 | +0.001 | −0.002 |
|  | Scalar | 728.65 | 450 | 0.958 | 0.957 | 0.062 | 19.45 | 19 | 0.428 | −0.000 | −0.001 |
| Astana vs Karaganda + Petropavlovsk | Configural | 736.44 | 412 | 0.950 | 0.944 | 0.070 |  |  |  |  |  |
|  | Metric | 762.17 | 431 | 0.949 | 0.945 | 0.069 | 25.72 | 19 | 0.138 | −0.001 | −0.001 |
|  | Scalar | 784.54 | 450 | 0.948 | 0.947 | 0.068 | 22.37 | 19 | 0.266 | −0.001 | −0.001 |
| Three study sites | Configural | 1007.18 | 618 | 0.941 | 0.933 | 0.077 |  |  |  |  |  |
|  | Metric | 1061.18 | 656 | 0.938 | 0.935 | 0.076 | 54.00 | 38 | 0.045 | −0.002 | −0.001 |
|  | Scalar | 1170.58 | 694 | 0.927 | 0.927 | 0.080 | 109.39 | 38 | <0.001 | −0.011 | +0.004 |

Multi-group maximum likelihood estimation with a mean structure; scale set by fixing factor variances to 1 in the reference group. Invariance was judged on the likelihood ratio test together with ΔCFI ≤ 0.010 and ΔRMSEA ≤ 0.015. Full scalar invariance held across the two random halves and between Astana and the two other sites pooled. Across the three sites, metric invariance held but scalar invariance did not, so item intercepts differ between sites and mean burnout levels are not directly comparable between cities.

**Supplementary Table S5** Complete standardized coefficients for the final hierarchical models (N = 323)

| **Predictor** | **EE β [95% CI]** | **SE** | **DP β [95% CI]** | **SE** | **PA β [95% CI]** | **SE** | **VIF** |
| --- | --- | --- | --- | --- | --- | --- | --- |
| Age (years) | 0.003 [−0.167, 0.172] | 0.086 | −0.019 [−0.227, 0.189] | 0.106 | 0.094 [−0.159, 0.348] | 0.129 | 6.45 |
| Length of service (years) | 0.013 [−0.153, 0.178] | 0.084 | 0.010 [−0.193, 0.213] | 0.103 | −0.153 [−0.400, 0.095] | 0.126 | 6.14 |
| Married (vs single) | −0.091 [−0.202, 0.020] | 0.056 | −0.030 [−0.166, 0.106] | 0.069 | 0.065 [−0.101, 0.231] | 0.084 | 2.76 |
| Divorced (vs single) | −0.051 [−0.144, 0.043] | 0.047 | −0.038 [−0.152, 0.077] | 0.058 | 0.030 [−0.109, 0.170] | 0.071 | 1.95 |
| Widowed (vs single) | −0.039 [−0.126, 0.048] | 0.044 | −0.040 [−0.147, 0.066] | 0.054 | 0.063 [−0.067, 0.193] | 0.066 | 1.69 |
| Number of children | 0.011 [−0.079, 0.102] | 0.046 | 0.049 [−0.062, 0.159] | 0.056 | −0.008 [−0.143, 0.127] | 0.069 | 1.83 |
| STEM (vs primary grades) | 0.012 [−0.072, 0.095] | 0.042 | 0.011 [−0.091, 0.113] | 0.052 | −0.118 [−0.243, 0.007] | 0.064 | 1.56 |
| Languages/humanities (vs primary grades) | 0.020 [−0.066, 0.106] | 0.044 | 0.001 [−0.104, 0.106] | 0.054 | 0.018 [−0.111, 0.147] | 0.065 | 1.66 |
| Arts/PE/technology (vs primary grades) | −0.050 [−0.127, 0.027] | 0.039 | −0.015 [−0.110, 0.079] | 0.048 | 0.046 [−0.070, 0.161] | 0.059 | 1.33 |
| Employment type | 0.055 [−0.025, 0.136] | 0.041 | 0.043 [−0.055, 0.142] | 0.050 | 0.080 [−0.040, 0.200] | 0.061 | 1.45 |
| Weekly teaching hours | 0.001 [−0.087, 0.088] | 0.045 | −0.031 [−0.139, 0.076] | 0.055 | −0.040 [−0.172, 0.091] | 0.067 | 1.72 |
| Total weekly workload | 0.044 [−0.030, 0.118] | 0.038 | 0.031 [−0.060, 0.122] | 0.046 | −0.079 [−0.191, 0.032] | 0.057 | 1.24 |
| Administrative duties | 0.046 [−0.028, 0.121] | 0.038 | 0.040 [−0.052, 0.131] | 0.046 | **0.140*** [0.028, 0.251] | 0.057 | 1.25 |
| Documentation load | 0.026 [−0.059, 0.111] | 0.043 | 0.013 [−0.091, 0.117] | 0.053 | 0.062 [−0.065, 0.189] | 0.065 | 1.62 |
| Daily screen time | 0.014 [−0.064, 0.092] | 0.040 | −0.026 [−0.121, 0.070] | 0.048 | 0.101 [−0.015, 0.217] | 0.059 | 1.36 |
| Information overload | 0.067 [−0.032, 0.166] | 0.050 | 0.043 [−0.079, 0.164] | 0.062 | 0.045 [−0.103, 0.193] | 0.075 | 2.20 |
| Conflict frequency | 0.062 [−0.021, 0.145] | 0.042 | **0.268***** [0.167, 0.370] | 0.052 | 0.002 [−0.123, 0.126] | 0.063 | 1.54 |
| Perceived social isolation | 0.032 [−0.050, 0.115] | 0.042 | **0.117*** [0.015, 0.218] | 0.051 | 0.005 [−0.119, 0.129] | 0.063 | 1.53 |
| Interpersonal climate | −0.066 [−0.139, 0.007] | 0.037 | **−0.131**** [−0.220, −0.041] | 0.046 | **0.135*** [0.026, 0.245] | 0.056 | 1.20 |
| Appraisal of digitalization | 0.001 [−0.072, 0.074] | 0.037 | 0.006 [−0.084, 0.096] | 0.046 | **0.155**** [0.045, 0.264] | 0.056 | 1.20 |
| Sleep duration | −0.031 [−0.105, 0.043] | 0.038 | 0.012 [−0.079, 0.103] | 0.046 | 0.084 [−0.027, 0.195] | 0.056 | 1.23 |
| Physical activity | **−0.105**** [−0.177, −0.033] | 0.037 | 0.019 [−0.069, 0.108] | 0.045 | 0.010 [−0.098, 0.117] | 0.055 | 1.16 |
| Meal irregularity | 0.049 [−0.024, 0.123] | 0.037 | 0.036 [−0.055, 0.126] | 0.046 | −0.044 [−0.154, 0.067] | 0.056 | 1.22 |
| Perceived work stress | **0.320***** [0.219, 0.422] | 0.052 | **0.239***** [0.114, 0.363] | 0.063 | 0.039 [−0.113, 0.191] | 0.077 | 2.31 |
| Emotional tension | **0.087*** [0.002, 0.171] | 0.043 | 0.069 [−0.034, 0.172] | 0.052 | **−0.128*** [−0.254, −0.002] | 0.064 | 1.59 |
| State anxiety (STAI-S) | **0.323***** [0.235, 0.411] | 0.045 | **0.126*** [0.018, 0.234] | 0.055 | **−0.291***** [−0.423, −0.159] | 0.067 | 1.74 |

*Model summaries.* EE: R² = 0.660, adjusted R² = 0.630, F(26,296) = 22.07, p < 0.001, Durbin-Watson 1.827. DP: R² = 0.488, adjusted R² = 0.443, F(26,296) = 10.85, p < 0.001, Durbin-Watson 1.965. PA: R² = 0.237, adjusted R² = 0.170, F(26,296) = 3.53, p < 0.001, Durbin-Watson 1.927. VIF values come from the emotional exhaustion model and are near-identical in the other two. The elevated values for age and length of service reflect their correlation of r = 0.90; scenario A of Supplementary Table S6 shows that removing length of service changes nothing. *p < 0.05; **p < 0.01; ***p < 0.001.

**Supplementary Table S6** Sensitivity analyses for the hierarchical models

| **Scenario** | **Outcome** | **Adjusted R²** | **Significant standardized coefficients** |
| --- | --- | --- | --- |
| **A. Length of service excluded** | EE | 0.631 | Work stress 0.320; state anxiety 0.323; emotional tension 0.087; physical activity −0.105 |
|  | DP | 0.445 | Conflict 0.268; work stress 0.238; state anxiety 0.126; isolation 0.117; climate −0.130 |
|  | PA | 0.168 | State anxiety −0.290; digitalization appraisal 0.161; administrative duties 0.135; emotional tension −0.132; climate 0.129 |
| **B. Adjusted for study site** | EE | 0.639 | Work stress 0.301; state anxiety 0.311; emotional tension 0.107; physical activity −0.084; Karaganda −0.283 |
|  | DP | 0.441 | Conflict 0.255; work stress 0.232; state anxiety 0.120; isolation 0.124; climate −0.140 |
|  | PA | 0.227 | State anxiety −0.289; digitalization appraisal 0.166; emotional tension −0.130; administrative duties 0.113; Petropavlovsk 0.629; Karaganda 0.490 |
| **C. State anxiety excluded** | EE | 0.566 | Work stress 0.433; emotional tension 0.123; physical activity −0.119; isolation 0.106; climate −0.083 |
|  | DP | 0.435 | Conflict 0.272; work stress 0.283; isolation 0.145; climate −0.137 |
|  | PA | 0.120 | Digitalization appraisal 0.178; emotional tension −0.161; climate 0.151; administrative duties 0.132 |
| **D. Blocks 1 to 3 only (appraised strain omitted)** | EE | 0.424 | Information overload 0.272; isolation 0.180; conflict 0.160; climate −0.149; meal irregularity 0.121; physical activity −0.115; sleep −0.099 |
|  | DP | 0.376 | Conflict 0.330; isolation 0.194; information overload 0.177; climate −0.180 |
|  | PA | 0.099 | Digitalization appraisal 0.185; climate 0.171; sleep 0.136; administrative duties 0.132 |

All coefficients standardized; only terms with p < 0.05 are listed. Every scenario retains the full block structure described in Section 2.5 except for the variable named. Scenario B replaces the site-adjusted analysis reported in the original submission. Scenario C addresses the content overlap between state anxiety and emotional exhaustion. Scenario D shows the demand and resource structure before appraised strain absorbs part of it.

**Supplementary Table S7** Exploratory stepwise regression models (N = 323)

| **Predictor** | **B** | **SE** | **β** | **95% CI (B)** | **t** | **p** | **VIF** |
| --- | --- | --- | --- | --- | --- | --- | --- |
| **Emotional exhaustion** (R² = 0.639; adjusted R² = 0.631; F(7,315) = 79.50; Durbin-Watson 1.850) | | | | | | | |
| Constant | −5.018 | 1.974 |  | −8.90 to −1.13 | −2.54 | 0.012 |  |
| Perceived work stress | 4.663 | 0.654 | 0.353 | 3.38 to 5.95 | 7.13 | <0.001 | 2.13 |
| State anxiety (STAI-S) | 0.345 | 0.042 | 0.346 | 0.26 to 0.43 | 8.28 | <0.001 | 1.52 |
| Physical activity | −1.423 | 0.409 | −0.120 | −2.23 to −0.62 | −3.48 | 0.001 | 1.03 |
| Information overload | 1.085 | 0.510 | 0.091 | 0.08 to 2.09 | 2.13 | 0.034 | 1.59 |
| Emotional tension | 1.139 | 0.520 | 0.089 | 0.12 to 2.16 | 2.19 | 0.029 | 1.43 |
| Total weekly workload | 1.144 | 0.553 | 0.072 | 0.06 to 2.23 | 2.07 | 0.040 | 1.06 |
| Conflict frequency | 1.065 | 0.526 | 0.079 | 0.03 to 2.10 | 2.03 | 0.044 | 1.34 |
| **Depersonalization** (R² = 0.472; adjusted R² = 0.463; F(5,317) = 56.56; Durbin-Watson 1.948) | | | | | | | |
| Constant | −0.402 | 1.568 |  | −3.49 to 2.68 | −0.26 | 0.798 |  |
| Perceived work stress | 2.088 | 0.364 | 0.301 | 1.37 to 2.80 | 5.73 | <0.001 | 1.65 |
| Conflict frequency | 2.053 | 0.333 | 0.291 | 1.40 to 2.71 | 6.16 | <0.001 | 1.34 |
| State anxiety (STAI-S) | 0.073 | 0.027 | 0.139 | 0.02 to 0.13 | 2.70 | 0.007 | 1.61 |
| Interpersonal climate | −0.896 | 0.343 | −0.111 | −1.57 to −0.22 | −2.61 | 0.010 | 1.09 |
| Perceived social isolation | 0.703 | 0.296 | 0.113 | 0.12 to 1.29 | 2.38 | 0.018 | 1.36 |
| **Personal accomplishment** (R² = 0.186; adjusted R² = 0.173; F(5,317) = 14.46; Durbin-Watson 1.864) | | | | | | | |
| Constant | 28.726 | 2.922 |  | 22.98 to 34.48 | 9.83 | <0.001 |  |
| State anxiety (STAI-S) | −0.213 | 0.037 | −0.309 | −0.28 to −0.14 | −5.82 | <0.001 | 1.10 |
| Appraisal of digitalization | 1.230 | 0.416 | 0.152 | 0.41 to 2.05 | 2.95 | 0.003 | 1.04 |
| Administrative duties | 1.808 | 0.638 | 0.145 | 0.55 to 3.06 | 2.83 | 0.005 | 1.03 |
| STEM (vs primary grades) | −2.209 | 0.961 | −0.117 | −4.10 to −0.32 | −2.30 | 0.022 | 1.01 |
| Interpersonal climate | 1.141 | 0.556 | 0.107 | 0.05 to 2.24 | 2.05 | 0.041 | 1.07 |

Stepwise selection (entry p ≤ 0.05, removal p ≥ 0.10) over the pool of occupational, psychoemotional, social-psychological, lifestyle, and digital-informational variables, with marital status and subject area dummy-coded. Age and length of service were examined correlationally only.

**Supplementary Table S8** Bootstrap stability of stepwise selection (500 resamples)

| **Predictor** | **EE (%)** | **DP (%)** | **PA (%)** |
| --- | --- | --- | --- |
| State anxiety (STAI-S) | 100.0 | 66.4 | 99.6 |
| Perceived work stress | 100.0 | 99.0 | 4.8 |
| Physical activity | 86.4 | 6.0 | 5.2 |
| Information overload | 58.8 | 17.2 | 16.8 |
| Emotional tension | 58.8 | 32.4 | 53.2 |
| Conflict frequency | 43.6 | 100.0 | 3.8 |
| Employment type | 36.8 | 9.4 | 20.6 |
| Interpersonal climate | 36.4 | 80.0 | 57.8 |
| Meal irregularity | 34.6 | 12.6 | 11.6 |
| Total weekly workload | 33.0 | 12.6 | 21.6 |
| Arts/PE/technology (vs primary grades) | 32.2 | 7.8 | 13.8 |
| Administrative duties | 31.2 | 23.8 | 74.8 |
| Married (vs single) | 26.4 | 5.2 | 3.8 |
| Sleep duration | 20.4 | 7.4 | 29.0 |
| Perceived social isolation | 19.0 | 68.0 | 2.8 |
| Documentation load | 15.4 | 10.8 | 23.2 |
| Daily screen time | 14.0 | 7.0 | 37.6 |
| Languages/humanities (vs primary grades) | 11.6 | 4.6 | 9.6 |
| Appraisal of digitalization | 9.2 | 6.0 | 87.8 |
| Weekly teaching hours | 9.0 | 6.8 | 8.0 |
| Divorced (vs single) | 5.8 | 7.6 | 5.2 |
| STEM (vs primary grades) | 5.4 | 2.4 | 59.0 |
| Number of children | 4.6 | 7.8 | 8.0 |
| Widowed (vs single) | 4.6 | 4.6 | 3.8 |

Percentage of 500 bootstrap resamples in which each predictor was retained by stepwise selection (entry p ≤ 0.05, removal p ≥ 0.10), using the same variable pool and dummy coding as Supplementary Table S8. Median model size: EE 8 (IQR 7 to 9; range 4 to 14); DP 6 (IQR 5 to 7; range 3 to 11); PA 7 (IQR 5 to 8; range 3 to 12). Fixed seed for reproducibility.
